# Genotype, mycorrhizae, and herbivory interact to shape strawberry plant functional traits

**DOI:** 10.3389/fpls.2022.964941

**Published:** 2022-10-26

**Authors:** Robert L. Whyle, Amy M. Trowbridge, Mary A. Jamieson

**Affiliations:** ^1^ Department of Biological Sciences, Oakland University, Rochester, MI, United States; ^2^ Department of Forest and Wildlife Ecology, University of Wisconsin-Madison, Madison, WI, United States

**Keywords:** biotic interactions, chemical ecology, genotype by environment, *Fragaria x ananassa*, herbivory, mycorrhizae, phenotypic plasticity, volatile organic compounds

## Abstract

Arbuscular mycorrhizal fungi (AMF) and herbivores are ubiquitous biotic agents affecting plant fitness. While individual effects of pairwise interactions have been well-studied, less is known about how species interactions above and belowground interact to influence phenotypic plasticity in plant functional traits, especially phytochemicals. We hypothesized that mycorrhizae would mitigate negative herbivore effects by enhancing plant physiology and reproductive traits. Furthermore, we expected genotypic variation would influence functional trait responses to these biotic agents. To test these hypotheses, we conducted a manipulative field-based experiment with three strawberry (*Fragaria x ananassa)* genotypes to evaluate plant phenotypic plasticity in multiple functional traits. We used a fully-crossed factorial design in which plants from each genotype were exposed to mycorrhizal inoculation, herbivory, and the combined factors to examine effects on plant growth, reproduction, and floral volatile organic compounds (VOCs). Genotype and herbivory were key determinants of phenotypic variation, especially for plant physiology, biomass allocation, and floral volatiles. Mycorrhizal inoculation increased total leaf area, but only in plants that received no herbivory, and also enhanced flower and fruit numbers across genotypes and herbivory treatments. Total fruit biomass increased for one genotype, with up to 30-40% higher overall yield depending on herbivory. Herbivory altered floral volatile profiles and increased total terpenoid emissions. The effects of biotic treatments, however, were less important than the overall influence of genotype on floral volatile composition and emissions. This study demonstrates how genotypic variation affects plant phenotypic plasticity to herbivory and mycorrhizae, playing a key role in shaping physiological and phytochemical traits that directly and indirectly influence productivity.

## Introduction

Plant functional trait responses are governed by genotypic variation and environmental conditions, of which biotic agents such as mycorrhizae and herbivores are key contributors (Kang Manjit et al., 1998; Agrawal, 2001; Barrett and Agrawal, 2004; Grinnan et al., 2013; Mathey et al., 2017). A wide range of plants are known to associate with arbuscular mycorrhizal fungi (AMF) in a symbiosis where fungi provide water and nutrients in exchange for carbohydrates (Bonfante and Anca, 2009; Kiers et al., 2011) and lipids (Keymer et al., 2017). Plant genotypes within the same species can exhibit variable responsiveness to AMF, especially depending on environmental conditions (Berger and Gutjahr, 2021 and citations within). Determining the genetic underpinnings and factors that augment or constrain favorable plant responses to environmental conditions remains a challenge, especially in the face of global change (Kang Manjit et al., 1998; Annicchiarico, 2002; Berg and Ellers, 2010; Tooker and Frank, 2012). Experimental designs evaluating multispecies interactions are required to determine how intraspecific variation governs phenotypic plasticity in response to above- and belowground mutualistic and antagonistic species interactions (Bardgett et al., 1998; van der Putten et al., 2001; Bardgett and Wardle, 2003; Tylianakis et al., 2008; Barber and Gorden, 2015).

Co-occurring interactions between plants, mycorrhizae and herbivores have important effects on plant functional traits and thus fitness (Vannette and Hunter, 2009; Barber and Gorden, 2015; Bennett and Meek, 2020). AMF can increase photosynthesis and stomatal conductance (Augé et al., 2016), above-ground biomass (Cordeiro et al., 2019), enhance plant reproduction (Barber and Gorden, 2015; Jacott et al., 2017; Golubkina et al., 2020; Kaur and Suseela, 2020), and potentially mitigate negative effects of herbivory through either enhanced tolerance or defense priming mechanisms (see Frew et al., 2022 and references therein). These fitness benefits have led to interest in developing AMF for improving ecological services in managed ecosystems and sustainable crop production (Berruti et al., 2016; Pagano et al., 2017; Basu et al., 2018). However, evidence supporting AMF inoculum application benefits remains mixed (Thirkell et al., 2017; Hart et al., 2018; Ryan and Graham, 2018). Factors influencing the benefits of AMF include intraspecific variation in the species involved (Bennett et al., 2006), abiotic environmental conditions (Dumbrell et al., 2010; Davison et al., 2021), and biotic environmental conditions such as mycorrhizal colonization and herbivores (Vannette and Hunter, 2009; Barto and Rillig, 2010; Schausberger et al., 2012; Barber, 2013; Wang et al., 2015; Orians et al., 2018). Understanding the ways in which AMF could help mitigate effects of herbivory and other biotic stressors is of special importance for developing sustainable and efficient management practices, especially in agricultural cropping systems (Basu et al., 2018).

While a number of studies have evaluated how herbivores and mycorrhizae interact to influence plant reproductive traits (Vannette and Hunter, 2009; Borowicz, 2010; Orians et al., 2018), research examining floral volatile organic compounds (VOCs) are notably absent. This lack of empirical evidence with regards to floral volatiles leaves researchers without a conceptual understanding of how AMF and herbivory together influence plant-pollinator interactions. Floral VOCs are critical components of pollinator recognition and attraction to flowers (Raguso, 2008; Burkle and Runyon, 2017). Additionally, these compounds are known to influence floral microbes that affect pollinator attraction and nutrition (Vannette, 2020) as well as floral herbivores (McCall and Irwin, 2006). Yet surprisingly, only one study, to our knowledge, has assessed how AMF influence floral VOC profiles and overall emissions. Becklin et al. (2011) found that plants with mycorrhizal associations exhibited reduced floral VOC production, suggesting that AMF could negatively affect pollination although this finding was context-dependent. Studies describing the effects of herbivory on floral VOCs are more numerous, and results similarly illustrate the importance of ecological context, with variable responses that depend on herbivore-plant species combinations, flower sex, and insect feeding modes (Effmert et al., 2008; Kessler and Halitschke, 2009; Theis et al., 2009; Schiestl, 2010; Pareja et al., 2012; Burkle and Runyon, 2016). Further studies are needed to understand how mycorrhizae and herbivores interact to shape plant functional traits, especially phytochemicals such as floral VOCs, which play a key role in plant fitness.

To address this knowledge gap, we examined the effects of AMF inoculation and herbivory on functional traits of the cultivated strawberry (*Fragaria* x *ananassa*), which is a major crop across the globe (Simpson, 2018). Using a field-based, potted-plant factorial experiment, we examined the effects of mycorrhizal spore inoculation and herbivory on phenotypic plasticity in strawberry plant physiology, reproduction, biomass allocation, and floral volatiles in three different strawberry cultivars, representing distinct genotypes. Given the importance of intraspecific variation in shaping multispecies interactions, we hypothesized that strawberry genotype would affect functional trait response to AMF inoculation and herbivory. We predicted that inoculated plants would exhibit greater gas exchange, growth and reproduction, and fluxes of floral volatiles. Further, we expected AMF inoculation to ameliorate some negative effects of herbivory.

## Materials and methods

### Plant material, mycorrhizal inoculation, and growing conditions

We used genetically identical clonal replicates of three *Fragaria* x *ananassa* cultivars (herein referred to as genotypes): ‘Seascape’, ‘Tribute’, and ‘Wasatch’. Replicate plants of each genotype were purchased as bare-root plants (Indiana Berry, Plymouth, IN, USA). In March 2019, these plants were potted in 9.1 L nursery fabric pots with a potting medium consisting of a 2:1:1 mixture of autoclave sterilized sphagnum peat moss (Lambert, Rivière-Ouelle, Québec, Canada), washed play sand (Kolorscape, Atlanta, GA, USA), and high-fired calcined clay (Turface, Buffalo Grove, IL, USA). We conducted mycorrhizal inoculations in mid-March using a commercial *Rhizophagus irregularis* inoculum (500 spores/gram; pure inoculum; Elite 91 Myco Jordan, Murietta, CA, USA). According to manufacturer instructions, mycorrhizal treatment plants received one teaspoon of powder inoculant onto the potting medium at the point of contact with roots and also dusted roots directly. Bareroot plants were likely colonized by mycorrhizae and other beneficial bacteria under nursery field conditions prior to our inoculation treatment. Previous studies, however, indicate that *R. irregularis* is an effective colonizer of strawberry roots (Todeschini et al., 2018). Furthermore, analyses of roots in this study indicated greater overall colonization of roots by fungal hyphae (mycorrhizae x genotype: F_2,82_ = 3.9, P = 0.020), vesicles (F_1,81_ = 12.9, P < 0.001), and arbuscules (F_1,82_ = 10.4, P = 0.002) in inoculated plants (**Supplementary Figure 1**).

Plants were arranged into replicated experimental blocks outdoors at the Oakland University Organic Student Farm located in Rochester, Michigan, USA (42°39’36.51”) in late April of 2019. Plants were watered weekly with ~1 L of water. Plants were initially grown without fertilizer for the first 3 weeks. Throughout the remainder of the experiment, all plants were fertilized once weekly by adding a complete nutrient solution formulated for soilless media (General Hydroponics, Santa Rosa, CA, USA) to water at half the manufacturer recommendations. We provided plants with a mixture of FloraGro (2-1-6 NPK), FloraBloom (0-5-4 NPK), and FloraMicro (5-0-1) for the given plant stage at the time of watering.

### Experimental design and herbivory treatment

Plants were randomly selected among genotypes to receive one of four treatments (control, herbivory, mycorrhizae, herbivory x mycorrhizae), in a randomized complete block design (n=12 individual plants of each genotype per treatment; **Supplementary Figure 2**). Treatment groups were repeated across 12 blocks, comprised of four sub-blocks. Each sub-block represented a treatment group and consisted of one plant from each genotype. Sub-blocks of plants receiving herbivory were spaced at least 1 m from sub-blocks without herbivory to reduce possible herbivory-induced volatile signaling (Šimpraga et al., 2016).

The herbivory treatment was carried out from mid-June to early July and consisted of two parts: (1) natural herbivory from four second and third instar larvae of *Vanessa cardui* (Carolina Biological Supply, Charlotte, NC, USA), a cosmopolitan, continuously breeding migrant butterfly, noted to be one of the most widespread, abundant, and polyphagous butterfly species worldwide (Scott, 1992; Janz, 2005). This species is a broad generalist that has been observed feeding on *Fragaria* spp., and it has been indicated as a potential pest of a variety of agricultural crops (e.g., Aslam and Wilde, 1991; Kelly and Debinski, 1999). Caterpillars were restricted to a single leaf per plant with a mesh bag for 6 days. (2) Simulated herbivory was carried out three days after caterpillars were removed from leaves and involved clipping leaf material to remove ~20-25% along with application of a 1 mM jasmonic acid solution in water (**Supplementary Figure 3**). Natural herbivory was followed by simulated herbivory on all plants in the two treatment groups receiving herbivory. Data for the herbivory period was collected only after both simulated and natural herbivory occurred. While this combination of experimental manipulations prevented us from determining if plant trait responses were due to tissue loss or induction of plant defenses, it provided a close approximation of natural herbivory *in situ* in such a way that leaf tissue damage could be standardized.(e.g., see van Kleunen et al., 2004 and Waterman et al., 2019).

### Measuring plant physiology, reproduction, and biomass

Physiological data were collected using a LI-COR 6800 portable photosynthesis system with head lamp attachment. Measurements were taken on clear days between 11:00 and 15:00. Photosynthesis (A) and stomatal conductance (g_s_) measurements were taken from the newest, fully expanded leaf on each plant. Samples were taken from undamaged leaves on plants that had received the herbivory treatment. Chamber environmental conditions were set to 800 mL/min air flow, 400 µmol/min CO_2_, 10,000 rpm fan speed, 65% relative humidity, and photosynthetically active radiation (PAR) set to 1000 µmol m^-2^ s^-1^ to simulate daytime sunlight levels. Temperature was set to ambient so that chamber temperature matched outside air temperature. Physiological measurements were taken during three time points: ‘pre-herbivory’, ‘post-herbivory’, and a post-herbivory ‘recovery’ period (**Supplementary Figure 3**).

We measured total flower and fruit number, and fruit weight (g), total leaf area (cm^2^), total plant dry weight (g), and root/shoot dry weights (g; data not shown). The number of flowers, number of fruits, and fruit weight were recorded twice weekly over the study period. Total leaf area was calculated at the end of the experiment using an allometric method developed by Demirsoy et al. (2005), in which the lengths of upper and left lobes of the largest and smallest strawberry leaves are used to calculate total leaf area. Initial plant fresh weight was recorded upon receiving bare-root plants and was included as a co-variate in statistical models. At the end of the study, we harvested and separated above- and belowground plant parts (flowers, fruits, leaves, roots). We gently washed potting media away from roots using water. All plant parts were dried at 60°C for 4 hours or as needed to record constant mass.

### Floral volatile collections

Floral volatiles were collected from new fully expanded flowers during two sampling periods: immediately after the herbivory treatment (‘Herbivory’ period) and several weeks later during a recovery period (‘Recovery’ period; **Supplementary Figure 3**). We simultaneously collected 16 floral and two blank samples for a four-hour period between 11:00 and 15:00 on clear days using a dynamic headspace sampling method similar to Burkle and Runyon (2016). We attempted to sample floral volatiles from an equal number of individuals from each treatment combination each day, though flower availability prevented a uniform number of individuals on several days, resulting in uneven replication among treatment combinations. Volatiles were sampled by enclosing flowers in 12 oz. polyethylene terephthalate cups with dome lids (Comfy Package, Rikkel Corp, NY, USA), pulling air through the semi-open system (**Supplementary Figure 4**). A 1/8” hole was drilled near the of the cup to maintain positive pressure and gas exchange. We used a flow rate of 200 mL/min to pull samples through HayeSep Q volatile traps (30 mg of adsorbent; VAS, Rensselaer, NY, USA) using either a Portable Volatile Assay System (PVAS22 model, Rensselaer, NY, USA) or an Air Lite low-flow air sample pump (SKC, Eighty Four, PA, USA). Average air temperature over sampling time was recorded using data from the Oakaland University weather station (weather station ID: KMIROCHE95). We recorded pump type for each sample and included it as a random effect in mixed effect general linear models. Prior tests indicated that these collection systems were functionally equivalent. We eluted traps with 200 µL of hexane into GC vials containing microinserts and stored samples at 20°C prior to chemical analyses. After VOC collections, flowers were cut at the base of the receptacle, dried for 48 hrs at 60°C and weighed.

### Chemical analyses

Floral volatiles were analyzed on an Agilent 7890A gas chromatograph (GC) with an Agilent 5977B mass spectrometer (MS) and HP-5 column (30 m x 250 µm x 0.25 µm) with helium as a carrier gas. The GC oven was maintained at 40°C for 1.5 min, then increased by 5°C/min to 175°C and 25°C/min to a final temperature of 280°C which was held for 2 min. Standards for trans-2-hexen-1-al, α-pinene, benzaldehyde, 6-methyl-5-hepten-2-one, cis-3-hexanyl acetate, D-limonene, benzyl alcohol, ocimene isomers, nonanal, p-anisaldehyde, trans-ß-ionone, and the internal standard, nonyl acetate, were purchased from Sigma-Aldrich (St. Louis, MO, USA). The internal standard was added at 9% v/v concentration to all hexane blanks, standards, and sample elution aliquots for GC analysis. Compounds were identified using NIST 08 Mass Spectral Search Program (National Institute of Standards and Technology, Gaithersburg, MD, USA) and confirmed by comparing retention times and mass spectra with commercial standards when available.

Volatiles were quantified by normalizing peak areas by the internal standard and applying the compound-appropriate four-point standard curve. In cases where samples contained volatiles for which we did not have standards, compounds were quantified as equivalents of compounds with similar functional groups. Peaks were integrated using Agilent’s Agile 2 integration software. If multiple peaks coeluted, manual drop-down integration was used. Quantities were then normalized by dry flower mass and converted to hourly emission rates. Strawberries produced three ocimene isomers, but due to some emissions being below the limit of detection and issues with co-eluting peaks, these isomers were analyzed collectively as ocimene isomers.

### Measuring root colonization by mycorrhizal fungi

To verify the effectiveness of our mycorrhizal inoculation, we measured AMF root colonization as % hyphal, vesicular, and arbuscular structures per root length. Specifically, dried roots were transferred to tissue cassettes, cleared for 5 mins in 3% KOH, acidified for 30 mins in 2% HCl, and stained for 20 mins in 0.05% trypan blue solution (methods described in Phillips and Hayman, 1970). Roots were scored for AMF colonization using the magnified intersection method (McGonigle et al., 1990) at 100 intersections per root.

### Statistical analyses

All statistical analyses were conducted using R Statistical Software (v4.1.2; R Core Team, 2021). We investigated treatment effects and their interactions on physiological traits, morphological traits, floral volatile emission rates, and colonization rates by fitting response variables to linear mixed models with block as a random effect followed by three-way ANOVA tests, and Tukey HSD tests to determine pairwise differences. Additionally, floral volatile emission models included pump ID as a random effect. Air temperature at sampling time of physiology measurements, initial plant weight, and average air temperature during volatile collections, were included as covariates in physiological, morphological, and volatile models, respectively. Least square means are reported.

Treatment effects on floral volatile composition were tested using three-way MANOVAs (‘adonis’ in vegan package in R). The ‘betadisper’ function (‘vegan’ package in R) was used to evaluate differences in volatile dispersion. Non-metric multidimensional scaling (NMDS) was used to visualize treatment effects on composition and dispersion. We used similarity percentage analysis (‘simper’ function in R) to determine which compounds contributed most to differences in significant treatment effects.

In addition to total volatile class emission rates, we analyzed the top five terpenes, top two benzenoid, and top two aliphatic compounds that contributed to differences among significant effects based on similarity percentage analysis results. These compounds included ocimene isomers, 6-methyl-5-hepten-2-one, α-farnesene, D-limonene, α-pinene, benzaldehyde, p-anisaldehyde, cis-3-hexanyl acetate, and (E)-3-hexen-1-ol. All emission rates were natural log-transformed to meet assumptions of normality. The long-chained aldehydes hexenal, heptenal, octanal, nonanal, and decanal were removed from analyses due to high variability in ambient background volatile collections (i.e. blank samples), making it difficult to reliably quantify emissions of these compounds from flowers in the field. Additionally, the sesquiterpenes α-copaene and ß-bourbonene were excluded from analyses as they were detected in less than a third of samples.

## Results

### Effect of genotype and biotic treatments on plant physiology, reproduction, and biomass

**Figure 1:**
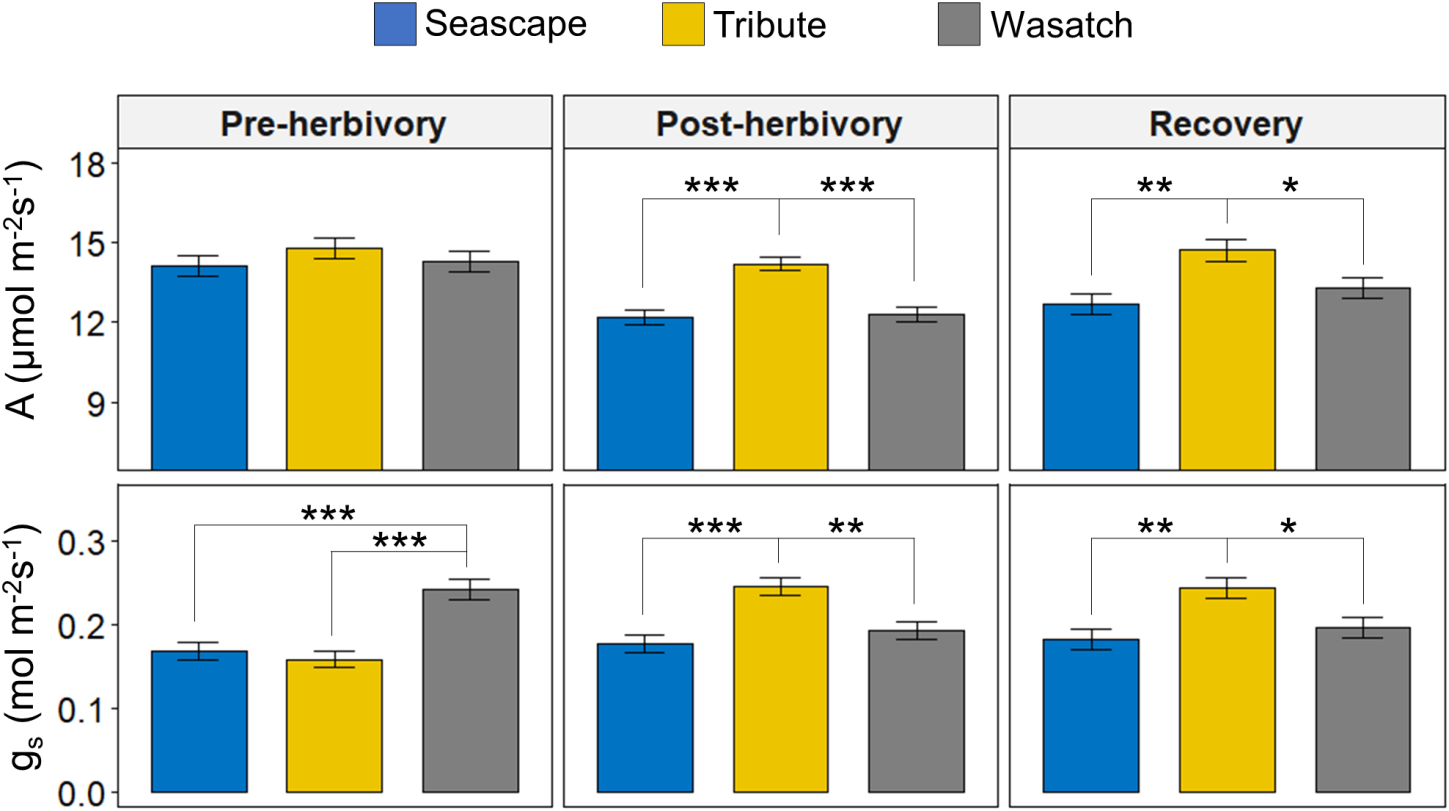
Photosynthesis (A) and stomatal conductance (g_s_) of Seascape (Pre-herbivory:n = 39; Post-herbivory and Recovery: n = 48), Tribute (Pre-herbivory: n = 39; Post-herbivory and Recovery: n = 48), and Wasatch (Pre-herbivory: n = 37; Post-herbivory and Recovery: n = 48). Physiological measurements were collected pre-herbivory (mid-May), post-herbivory (early-July), and after an herbivory recovery period (mid-August). Bars are least-square means ± 1SE. Brackets with an asterisk represent significant pairwise differences (P ≤ 0.05) as determined by Tukey’s HSD tests. * = <0.05, ** = <0.01, *** = <0.001.

Genotype was the primary determinant of variation in photosynthesis (A) and stomatal conductance (g_s_) (**Figure 1**; **Table 1**). Prior to herbivory treatments, there were no significant differences in A among genotypes and g_s_ was approximately 30% higher in Wasatch than in other genotypes. During the post-herbivory and recovery sampling periods, Tribute exhibited higher A and g_s_ relative to Seascape and Wasatch. In recovery measurements, plants receiving herbivory exhibited 16% higher conductance rates (**Table 1**).

**Table 1:** Mixed model ANOVA results showing effects on plant physiological measurements: Photosynthesis (A) and stomatal conductance (g_s_).

Treatments	Pre-herbivory		Post-herbivory		Recovery	
	A	g_s_	A	g_s_	A	g_s_
Mycorrhizae	0.079	1.193	3.056	0.422	2.277	0.399
Herbivory	NA	NA	1.265	0.132	1.790	4.67*
Genotype	0.866	15.70***	21.80***	10.62***	7.109**	5.73**
Mycorrhizae*Herbivory	NA	NA	0.895	1.608	0.025	0.368
Mycorrhizae*Genotype	0.766	0.956	0.422	0.242	0.243	0.272
Herbivory*Genotype	NA	NA	0.265	0.489	0.468	1.263
Temperature	2.847	50.22***	21.28***	0.012	9.790**	9.02**

NAs indicate when variables or covariates were not used in models. Significant F values are in bold: *P ≤ 0.05, **P ≤ 0.01, ***P ≤ 0.001. Temperature at the time of measurement was used as a covariate.

**Figure 2:**
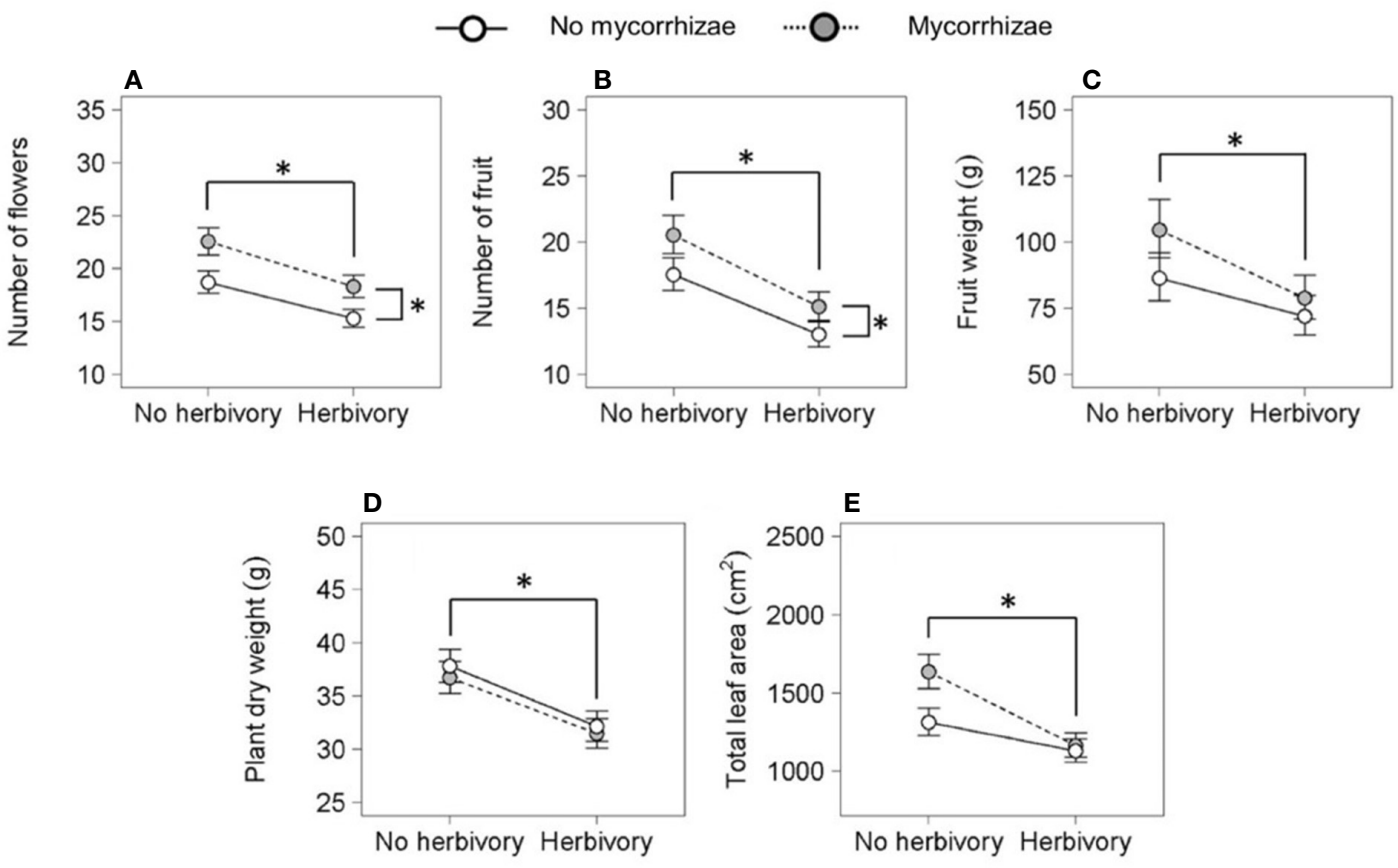
Key effects of herbivory and mycorrhizal inoculation treatments (n = 36) on the number of flowers **(A)** and fruit **(B)**, fruit weight **(C)**, plant dry weight **(D)**, and total leaf area **(E)**. Asterisks with brackets indicate significant main effects (P < 0.05). Asterisks above data points indicate herbivory effects and asterisks on the right indicate mycorrhizal inoculation effects. Values are least-square means ± 1 SE.

**Figure 3:**
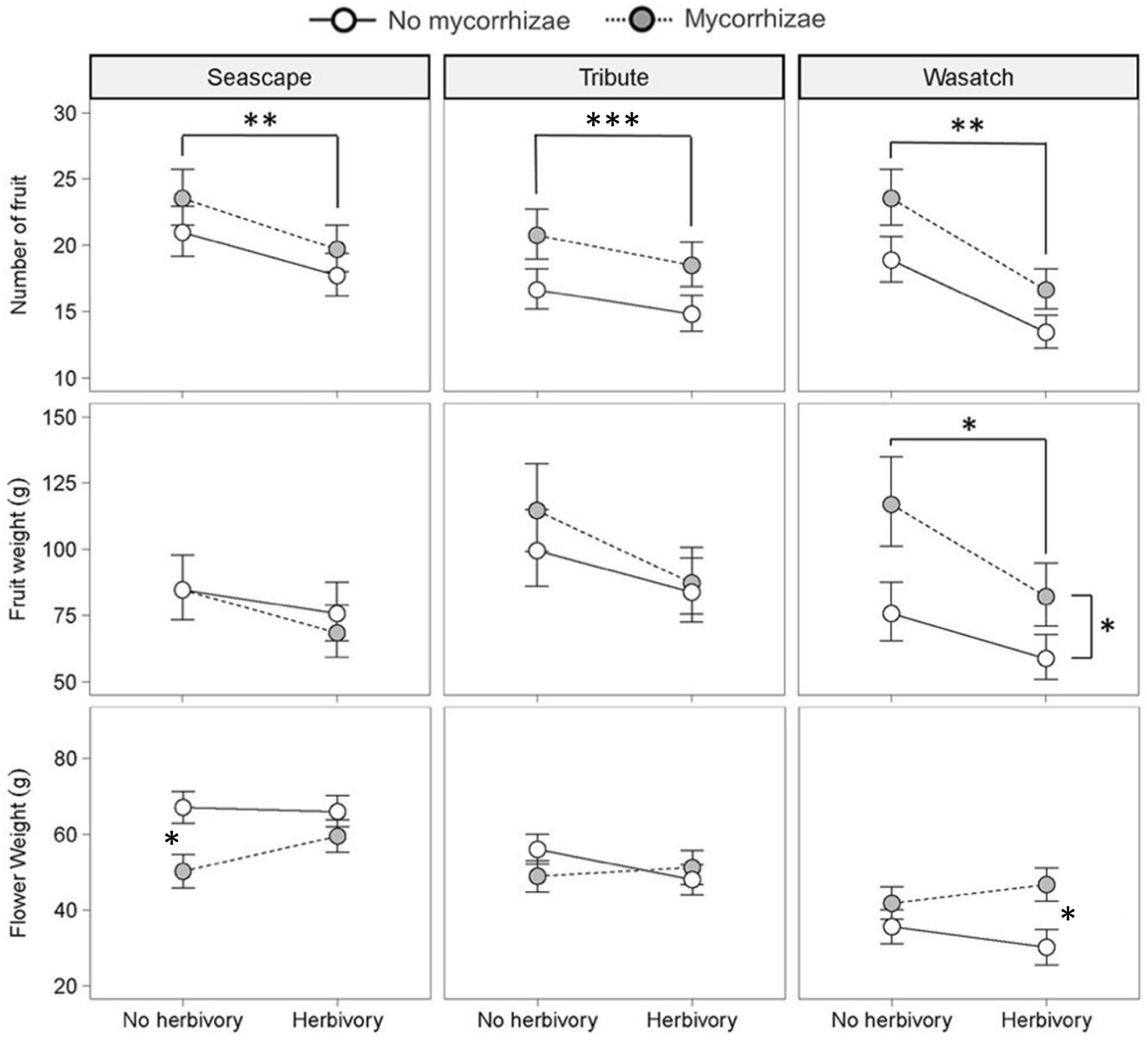
Key effects of herbivory and mycorrhizal inoculation treatments on plant allocation patterns within genotypes (n = 12). Asterisks with brackets indicate significant main effects and asterisks without brackets indicate significant pairwise differences from *post hoc* analyses (P ≤ 0.05). Values are least-square means ± 1SE. * = <0.05, ** = <0.01, *** = <0.001.

Reproductive traits were influenced by genotype and both biotic treatments. Genotypes differed in total flower and fruit number and flower dry weight (**Figure 3**; **Table 2**). Herbivory explained the greatest amount of variation in strawberry reproductive traits. Specifically, the herbivory treatment reduced fruit weight by 20% and total flower and fruit number by 19% and 25%, respectively. (**Figures 2A–C**; **Table 2**). Mycorrhizal inoculation enhanced flower number by 20% and fruit number by 17% (**Figures 2A, B**; **Table 2**). Mycorrhizal inoculation also benefited the total fruit yield (measured by weight), although that effect was genotype-specific (**Figure 3**; **Table 2**). Namely, Wasatch responded most strongly to the AMF treatment, with 30-40% greater fruit production overall. Total fruit weight produced by Wasatch over the growing season increased by 42% when grown in the mycorrhizal treatment with no herbivory. Even when exposed to herbivory, mycorrhizal inoculation enhanced fruit production by 34% on average (**Figure 3**; **Table 2**). We found no interactive treatment effects of herbivory and mycorrhizae on strawberry reproductive traits.

**Table 2:** Mixed model ANOVA results examining effects on plant biomass allocation response variables.

Treatments	Flower dry weight	Total flower number	Total fruit weight	Total fruit number	Total dry weight	Total leaf area
Mycorrhizae	0.144	10.043**	3.100	5.773*	0.521	3.687
Herbivory	0.001	13.472***	8.107**	22.214***	17.281***	17.137***
Genotype	21.584***	2.864	2.443	15.894***	89.372***	94.810***
Mycorrihzae*Herbivory	3.746	0.008	0.418	0.002	0.021	2.508
Mycorrhizae*Genotype	5.878**	0.356	2.494	0.493	1.305	0.514
Herbivory*Genotype	0.600	1.413	0.272	0.408	0.829	2.453
IPW	6.937	30.655***	1.279	6.967**	11.408***	5.152*

NA indicates when covariates were not included in model. Bolded F values are significant: * P < 0.05; ** P < 0.01; *** P < 0.001. Initial plant weight (IPW) was included in models as a covariate in all models except for % root length colonized, in which root dry weight was included as a covariate.

Genotype and herbivory were key predictors of variation in strawberry total plant dry weight and leaf area. Wasatch root and shoot dry weights were ~40% larger and had over twice the leaf area of Seascape and Tribute plants by the end of the study. Similar to reproductive traits, herbivory negatively impacted all biomass metrics. Herbivory reduced total dry weight by 15% (**Figure 2D**; **Table 2**), which was partitioned into a 13% decrease in root weight (F_1,119_ = 12.2, P = <0.001) and 21% decrease in shoot dry weight (F_1,121_ = 20.5, P = <0.001; see **Figure 2D** for total dry weight effects) across genotypes. However, there were no significant effects on root:shoot ratio (data not shown). Herbivory reduced final total leaf area of all genotypes equally by ~20% (**Figure 2E**; **Table 2**).

### Effect of genotype and biotic treatments on floral volatile emissions

**Figure 4:**
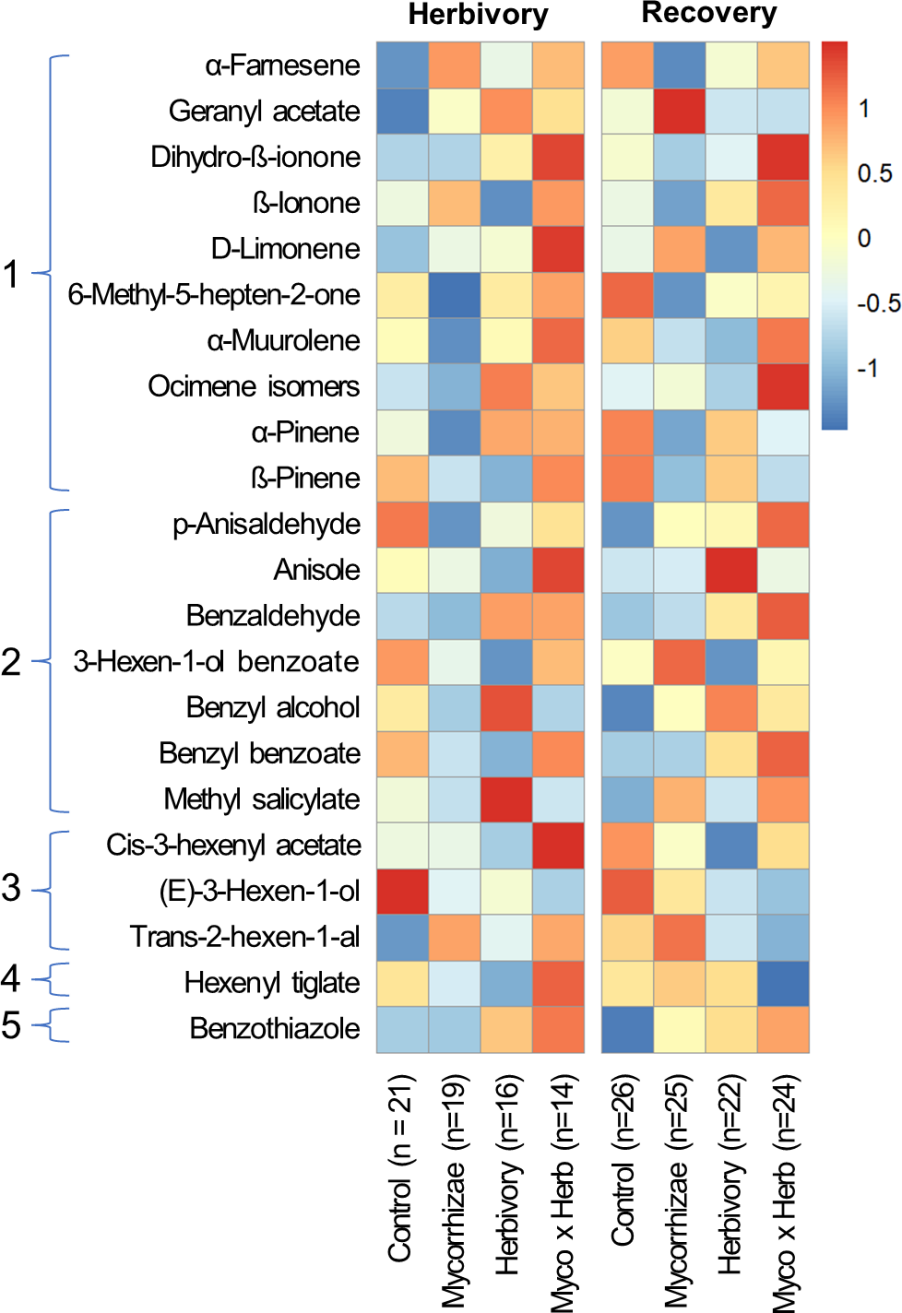
Heatmap of floral volatile emissions for strawberry plants in each treatment group during the ‘herbivory’ (left) and the ‘recovery’ (right) volatile sampling periods. Colors illustrate relative differences in floral volatile emissions as determined by range-scaled log-transformed values of VOC emission rates (ng/g of dry flower/hr) for each compound. Compounds are grouped by volatile class: (1) terpenes, (2) benzenoids, (3) aliphatic compounds, (4) C5 branched-chain compounds, and (5) S-containing compounds.

**Figure 5:**
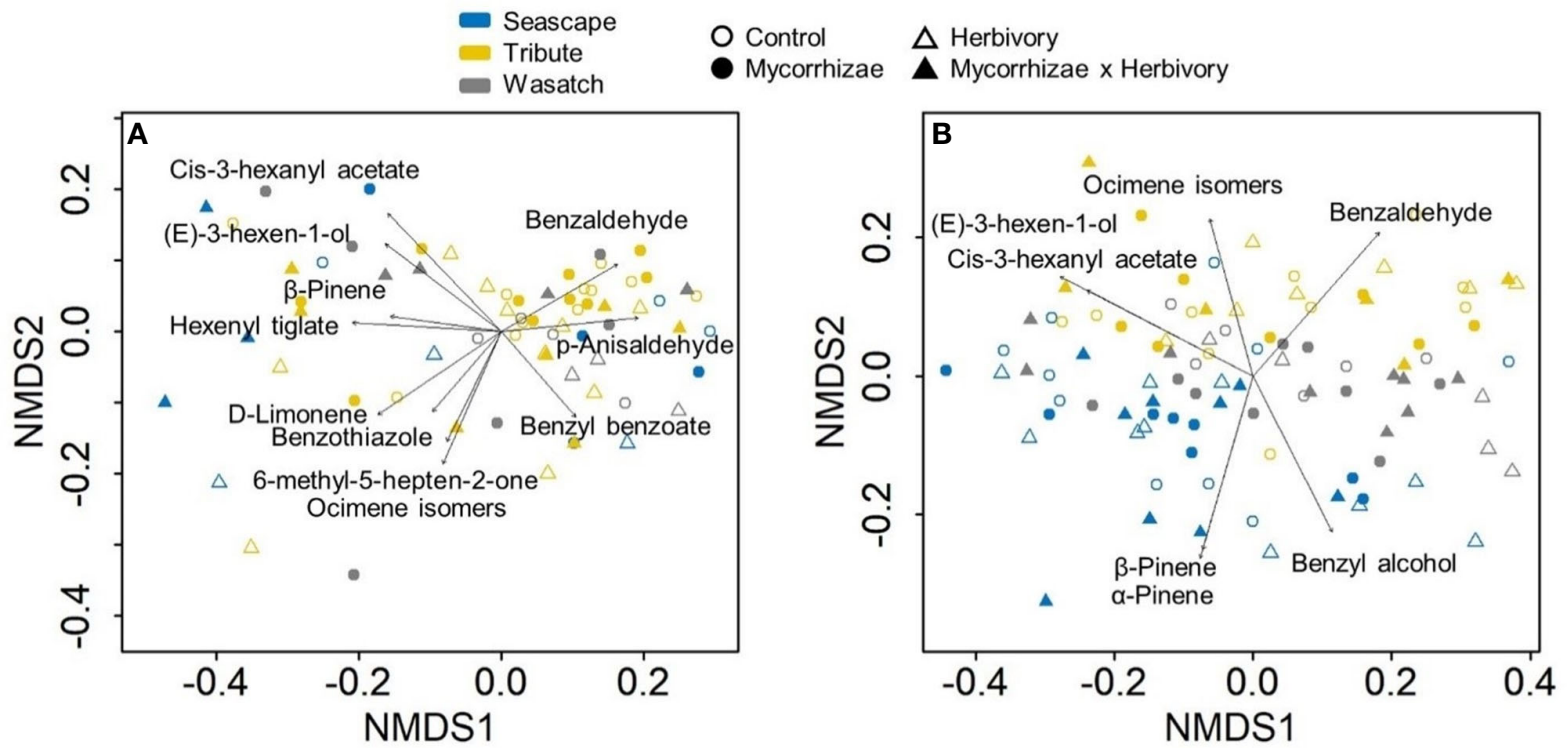
Non-metric multidimensional scaling (NMDS) plots for herbivory **(A)** and recovery **(B)** volatile sampling periods. Plots illustrate variation in floral volatile composition for plants of three strawberry genotypes (Seascape, Tribute, and Wasatch) grown in one of four treatments (control, mycorrhizae, herbivory, and herbivory x mycorrhizae). Sample sizes (n) of genotype and treatment combinations ranged from 3 to 11 for a total of 70 plants during the herbivory period. Recovery period sample sizes range from 6 to 10 for a total of 97 plants.

**Figure 6:**
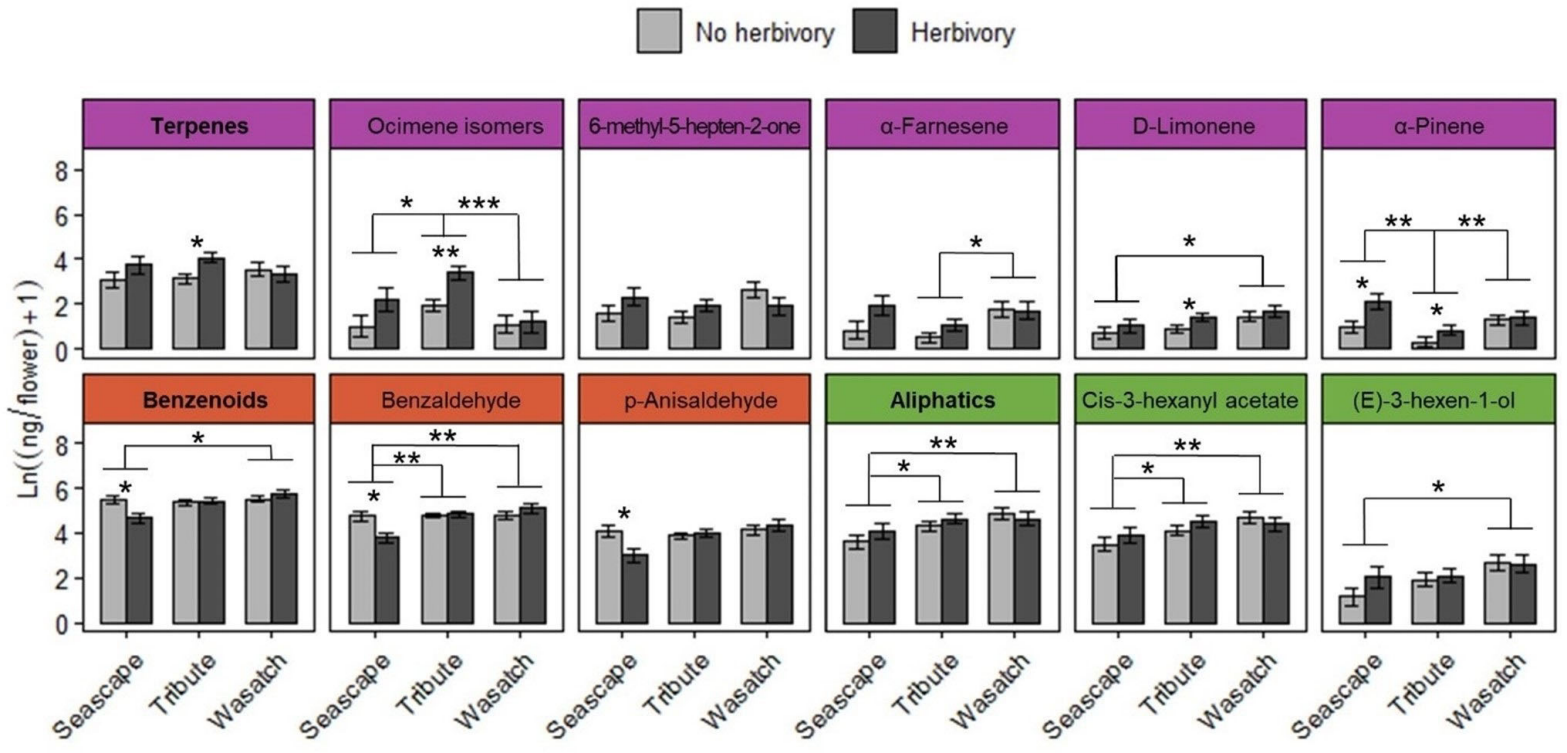
Summary of floral volatiles collected after the herbivory treatment period across genotypes. Total terpene (purple), benzenoid (red), and aliphatic (green) emissions are bolded. Graphs for individual compounds are colored according to their compound class. Asterisks above brackets indicate significant pairwise differences among genotypes (P ≤ 0.05). Asterisks below brackets indicate significant pairwise differences among herbivory treatments within genotypes (P ≤ 0.05). Sample sizes (n) of genotype and herbivory treatment combinations range from 6 (Seascape, No herbivory) to 21 (Tribute, no herbivory) plants. * = <0.05, ** = <0.01, *** = <0.001.

Flowers from all genotypes consistently produced 24 common volatile compounds, including 12 terpenoid, 3 aliphatic, 7 benzenoid, one S-containing, and one C5 branched-chain compound (**Figure 4**; **Supplementary Table 1**). Similar to other reproductive traits, genotype had a stronger overall influence on variation in floral volatile emission rates and composition relative to biotic treatments (**Figures 5**, **6**; **Tables 3**, **4**). Biotic treatments primarily affected volatile emission rates during the ‘herbivory’ sampling period, and treatment effects were less apparent during the ‘recovery’ period (**Figures 4**–**6**; **Tables 3**, **4**). Temperature positively influenced emission rates of all compound classes except aliphatics during the ‘herbivory’ period, and all but benzenoids and aliphatic compounds during the ‘recovery’ period (**Table 3**). Flower dry weight was generally positively associated with all volatile compound classes (**Table 3**).

**Table 3:** Mixed model ANOVA results evaluating effects on floral volatile organic compound classes.

Treatments	Total Emissions	Total Terpenes	Total Benzenoids	Aliphatic compounds	C-5 branched-chain compounds	S-containing compound
**Herbivory Period**						
Mycorrhizae	0.208	0.099	1.205	0.130	0.662	0.006
Herbivory	0.708	7.045*	0.692	1.268	2.619	2.671
Genotype	4.249*	0.224	2.776	5.945**	4.850*	0.984
Mycorrhizae*Herbivory	3.520	0.154	1.754	0.735	0.719	0.053
Mycorrhizae*Genotype	0.008	0.414	1.267	1.811	2.588	1.131
Herbivory*Genotype	2.994	2.312	4.912*	1.005	0.913	0.331
Temperature	0.114	9.350**	4.357*	0.007	4.078*	38.779***
Flower dry weight	43.600***	22.742***	4.912*	41.286***	20.825***	13.219***
**Recovery Period**						
Mycorrhizae	1.933	2.691	0.452	2.237	0.233	2.100
Herbivory	1.287	1.939	2.893	1.330	0.774	2.830
Genotype	2.770	1.804	6.902**	0.594	0.632	0.285
Mycorrhizae*Herbivory	0.001	0.606	0.390	0.280	0.150	0.071
Mycorrhizae*Genotype	2.100	0.829	1.737	0.285	2.531	0.326
Herbivory*Genotype	2.181	1.459	1.264	0.182	0.350	2.232
Temperature	6.784*	4.386*	2.527	1.876	5.001*	15.917***
Flower dry weight	48.805***	36.982***	12.411***	32.815***	9.899**	2.993

Significant F values are in bold: * P < 0.05, ** P < 0.01, *** P < 0.001. Temperature at the beginning of collection was included in models as a covariate.

**Table 4:** Mixed model ANOVA results examining effects on individual floral volatile organic (VOC) compounds.

	Terpenes					Benzenoids		Aliphatics	
Treatments	α-farnesene	D-limonene	6-methyl-5-hepten-2-one	ocimene	α-pinene	benzaldahyde	p-anisaldehyde	cis-3-hexanyl acetate	(E)-3-hexen-1-ol
***Herbivory Period***									
Mycorrhizae	0.001	1.346	0.016	0.030	0.537	0.378	0.848	0.365	0.091
Herbivory	4.084*	5.596*	1.131	12.702***	8.133**	0.634	0.996	1.663	1.089
Genotype	4.542*	3.339*	2.172	8.261***	8.198***	3.875*	2.271	5.596**	4.492*
Mycorrhizae*Herbivory	0.081	0.661	0.130	0.006	1.602	0.911	1.615	0.836	0.378
Mycorrhizae*Genotype	1.078	2.279	0.638	0.634	1.208	1.692	1.367	1.750	0.380
Herbivory*Genotype	1.144	0.267	2.613	1.780	1.454	5.895**	4.317*	1.227	0.738
Temperature	2.669	13.021***	0.084	10.013**	0.579	**3.317** ^†^	2.493	0.005	0.035
Flower dry weight	15.611***	29.564***	16.859***	9.303**	23.085***	7.698**	1.242	40.696***	27.091***
***Recovery Period***									
Mycorrhizae	0.005	2.443	0.005	0.000	0.401	0.032	0.830	2.571	0.455
Herbivory	0.591	0.126	0.390	1.003	0.992	1.963	0.268	1.093	1.390
Genotype	4.985**	0.342	0.835	24.052***	51.153***	18.076***	0.883	0.320	1.440
Mycorrhizae*Herbivory	1.851	0.010	0.496	1.002	0.036	0.000	0.087	0.577	0.118
Mycorrhizae*Genotype	1.642	0.921	0.340	1.688	0.113	1.859	2.475	0.235	0.776
Herbivory*Genotype	0.725	1.021	0.945	0.323	0.563	1.890	0.666	0.239	0.361
Temperature	1.796	0.056	1.848	5.222*	2.885	4.299*	3.420	1.313	12.933***
Flower dry weight	13.520***	5.816*	38.906***	29.281***	25.404***	12.563***	2.285	29.718***	34.600***

Temperature at the beginning of volatile collection and flower dry weight were included in models as a covariate. Significant F values are in bold: * P < 0.05, ** P < 0.01, *** P < 0.001.

During the ‘herbivory’ volatile collection period, the composition of strawberry flower volatile emissions differed significantly between genotypes across all treatment groups (F_2,57_ = 4.152, P = 0.001; **Figure 5A**). Similarity percentage analyses showed ocimene isomers, α-pinene, α-farnesene, and 6-methyl-5-hepten-2-one, and (E)-3-hexen-1-ol were responsible for differences among genotypes (**Supplementary Table 2**). Volatile compositions also differed between herbivory treatment and herbivory control plants (F_1,57_ = 3.245, P = 0.009; **Figure 5**). These compositional differences were largely driven by three compounds: ocimene isomers, (E)-3-hexen-1-ol, and 6-methyl-5-hepten-2-one (**Supplementary Table 2**).

Total floral volatile emissions differed by genotype during ‘herbivory’ collections (**Table 3**). Wasatch had the highest emission rate among genotypes, followed by Tribute, and was significantly higher than Seascape (**Table 3**). Herbivory increased total terpene emissions by 61% (**Figure 6**; **Table 3**). This effect was driven by several individual terpene compounds. Notably, herbivory increased ocimene isomers by ~200% (**Figure 6**). Herbivory affected benzenoid emissions differently among genotypes (**Table 3**), with no effect on Tribute and Wasatch but a 50% reduction in benzenoids compared to controls (**Table 3**). Aliphatic compounds differed among genotypes, as Seascape emitted significantly less than Tribute and Wasatch (**Figure 6**; **Table 4**).

During the ‘recovery’ collections, floral volatile composition differed only by genotype (F_2,85_ = 6.406, P = 0.001; **Figure 5B**), indicating that herbivore damage did not have long-lasting effects. Variation in volatile composition among genotypes was largely driven by α-pinene, ocimene isomers, and α-farnesene, and cis-3-hexanyl acetate (**Supplementary Table 3**). Surprisingly, Tribute flowers emitted 70% more benzenoids than Seascape (**Figure 6**; **Table 3**). There were no significant treatment effects on total emissions, terpenes, and aliphatic compound during ‘recovery’ collections.

## Discussion

Adaptive phenotypic plasticity is influenced by plant genotype, and thus shapes plant functional trait response to antagonistic and mutualistic species interactions (Agrawal, 2001). In this study, we found that genotype was the primary factor contributing to variation in strawberry plant physiological, reproductive, growth, and phytochemical traits. Moreover, genotype interacted with herbivory and mycorrhizae to yield cultivar-specific outcomes to biotic interactions, confirming our hypothesis that genotype would interact with biotic agents to influence trait expression. For example, while mycorrhizal inoculation improved the number of flowers and fruits in all strawberry genotypes and across herbivory treatments, the benefits of inoculation on total fruit yield were only found in one genotype, in which yields were increased by 30-40%. While AMF inoculation and herbivory treatments altered several growth, reproductive, and volatile traits, genotype produced greater effect sizes than treatments across the majority all plant traits. In this study, the Wasatch genotype demonstrated relatively higher mycorrhizal colonization compared to controls, which likely underlie some of the observed genotype x AMF interactive effects. Previous studies indicate that plant genotypes, including different strawberry cultivars, can show variable responsiveness to different species or strains of AMF (Chávez and Ferrera-Cerrato, 1990; Vestberg, 1992; Estaún et al., 2010; Sinclair et al., 2014), and recent research highlights the importance of fungal diversity (De Tender et al., 2021; Frew et al., 2022). Because we did not identify fungal species when measuring root colonization, there is uncertainty about AMF community composition and the extent to which our inoculation contributed to higher colonization.

AMF have been shown to alter plant reproductive traits such as increased flower and fruit number as well as fruit yield (Gange and Smith, 2005; Wolfe et al., 2005; Perner et al., 2007; Varga and Kytöviita, 2010). While increases in fruit yield as a result of mycorrhizal inoculation are often attributed to improvements in physiology and nutrient availability (Smith and Read, 2010), increases in flower number can be attributed to both physiological and hormonal changes that result from mycorrhizal associations (Bryla and Koide, 1998), leading to extended flowering periods and increases in the number of flowers (Torelli et al., 2000). In this study, we expected that inoculated plants would have improved trait expression as a result of improved physiology. However, we found inoculation had no effect on photosynthesis and stomatal conductance, yet inoculated strawberry plants produced more flowers and fruits than controls, indicating that the increased floral display could be a result of changes in plant signaling upon symbiosis rather than improved gas exchange. While many studies have found mycorrhizal plants exhibit improved gas exchange, others have found no effect or even reductions in the same measures, which can ultimately result in reduced yields (see Augé et al., 2016; Balestrini et al., 2020 and references therein). However, enhanced flower production can improve a plant’s attractiveness to pollinators (Bauer et al., 2017), and studies have shown that increasing pollinator abundance can improve strawberry yield, firmness, shelf-life, and nutritional content (Klatt et al., 2014). Our results suggest that even in the absence of physiological benefits, mycorrhizal inoculation may increase strawberry production indirectly by increasing the number of flowers produced by host plants, but benefits will still depend on cultivar selection.

As expected, herbivory had negative effects on the expression of nearly all growth and reproductive plant traits. Interestingly, herbivory altered floral VOC composition and emission rates immediately following treatment application, but effects were absent several weeks after herbivory, during the recovery period. Floral VOC compositional shifts were driven largely by an increase in terpene emissions, in particular of ocimene isomers. β-Ocimene plays a key role in plant tri-trophic interactions as it is emitted from plant vegetative tissues upon herbivore attack and is known to attract predators and parasitoids of herbivores (Loughrin et al., 1994; Miresmailli et al., 2010). Although there are no studies directly linking floral β-ocimene to increased pollinator activity, its spatial and temporal emission patterns resemble those of known bee attractants, suggesting that β-ocimene may be important for pollinator interactions (Farré-Armengol et al., 2017). Thus, it’s possible that increases in β-ocimene following herbivore attack could improve plant fitness by reducing herbivore abundance while simultaneously increasing pollinator attraction to flowers.

In addition to herbivore effects on volatile composition, all three genotypes exhibited significantly different compositions, driven largely by variation in α-farnesene, α-pinene, and ocimene isomers. Thus, both herbivory and genotypic effects on floral VOC composition have the potential to influence pollinator attraction to strawberry flowers. Klatt et al. (2013) and Ceuppens et al. (2015) found that differences in floral VOC profiles among three *Fragaria x ananassa* genotypes influenced attraction of bee pollinators such as *Osmia bicornis* and *Bombus impatiens* to strawberry genotypes. Further studies examining the effects of crop floral volatile profiles on pollinators, herbivores, florivores, and natural enemies are needed, as these biotic interactions influence plant fitness as well as crop yield and quality.

In summary, our findings indicate that genotype plays a key role in determining how herbivory and mycorrhizae influence strawberry functional trait expression. We found that AMF inoculation can mitigate negative consequences of herbivory to plant reproductive success, confirming our original hypothesis, but genotype will ultimately modulate plant response. Thus, the value of AMF, and likely other beneficial microbes, for enhancing crop production depend on genotype and cultivar selection. Here, we demonstrate that AMF inoculations have the potential to increase fruit yield by ~40%, which may be especially important for crop production systems lacking natural soil microbes, like those that involve soilless potting media supplied with nutrient solutions. In these systems, the addition of AMF may reduce nutrient inputs and losses. Furthermore, this study illustrates the importance of genotype and herbivore-induced phenotypic plasticity in understanding variation in floral volatiles emissions and composition, which is important not only for shaping plant interactions with pollinators (e.g. Klatt et al., 2013) but also plant-associated microbes (e.g. Wei et al., 2022). Developing a better understanding of how genotypic variability shapes phenotypic plasticity in functional traits, including phytochemicals that mediate species interactions, can help inform cultivar selection and selective breeding for sustainable agriculture.

## Data availability statement

The raw data supporting the conclusions of this article will be made available by the authors, without undue reservation.

## Author contributions

RW and MJ contributed to conception and design of the study. RW performed experimental protocols, GC-MS and statistical analyses with guidance from MJ and AT. RW wrote the first draft of the manuscript. RW, MJ, and AT wrote sections of the manuscript. All authors contributed to manuscript revision, read, and approved the submitted version.

## Funding

Research was funded by a Foundation for Food and Agricultural Research New Innovator Award to MJ (FFAR Award No. 430876) and Oakland University Provost Graduate Student Research Award to RW. These funders had no role in study design, data collection and analysis, decision to publish, or preparation of the manuscript.

## Acknowledgments

We thank Rob Raguso and Ken Keefover-Ring for assistance with floral VOC method development and Tom Raffel for statistical consultation.

## Conflict of interest

The authors declare that the research was conducted in the absence of any commercial or financial relationships that could be construed as a potential conflict of interest.

## Publisher’s note

All claims expressed in this article are solely those of the authors and do not necessarily represent those of their affiliated organizations, or those of the publisher, the editors and the reviewers. Any product that may be evaluated in this article, or claim that may be made by its manufacturer, is not guaranteed or endorsed by the publisher.

## References

[B1] Agrawal A. A. (2001). Ecology: Phenotypic plasticity in the interactions and evolution of species. Science 294, 321–326. doi: 10.1126/science.1060701 11598291

[B2] Annicchiarico P. (2002). Genotype X environment interactions: Challenges and opportunities for plant breeding and cultivar recommendations. Ed. Annicchiarico P. (Rome, Italy: Food and Agriculture).

[B3] Aslam M. Wilde G. E. (1991). Potential insect pests of sunflower in Kansas. J. Kansas Entomological Soc. 64, 109–112. Available at: https://www.jstor.org/stable/25085253.

[B4] Augé R. M. Toler H. D. Saxton A. M. (2016). Mycorrhizal stimulation of leaf gas exchange in relation to root colonization, shoot size, leaf phosphorus and nitrogen: A quantitative analysis of the literature using meta-regression. Front. Plant Science. 7, 1084. doi: 10.3389/fpls.2016.01084 PMC4965464 27524989

[B5] Balestrini R. Brunetti C. Chitarra W. Nerva L. (2020). Photosynthetic traits and nitrogen uptake in crops: Which is the role of arbuscular mycorrhizal fungi? Plants 9, 1–16. doi: 10.3390/plants9091105 PMC7570035 32867243

[B6] Barber N. A. (2013). Arbuscular mycorrhizal fungi are necessary for the induced response to herbivores by cucumis sativus. J. Plant Ecol. 6, 171–176. doi: 10.1093/jpe/rts026

[B7] Barber N. A. Gorden N. L. S. (2015). How do belowground organisms influence plant–pollinator interactions? J. Plant Ecol. 8, 1–11. doi: 10.1093/jpe/rtu012

[B8] Bardgett R. D. Wardle D. A. (2003). Herbivore-mediated linkages between aboveground and belowground communities. Ecology 84, 2258–2268. doi: 10.1890/02-0274

[B9] Bardgett R. D. Wardle D. A. Yeates G. W. (1998). Linking above-ground and below-ground interactions: How plant responses to foliar herbivory influence soil organisms. Soil Biol. Biochem. 30, 1867–1878. doi: 10.1016/S0038-0717(98)00069-8

[B10] Barrett R. D. H. Agrawal A. A. (2004). Interactive effects of genotype, environment, and ontogeny on resistance of cucumber (Cucumis sativus) to the generalist herbivore, spodopera exigua. J. Chem. Ecol. 30, 37–51. doi: 10.1023/B:JOEC.0000013181.50319.9d 15074656

[B11] Barto E. K. Rillig M. C. (2010). Does herbivory really suppress mycorrhiza? a meta-analysis. J. Ecol. 98, 745–753. doi: 10.1111/j.1365-2745.2010.01658.x

[B12] Basu S. Rabara R. C. Negi S. (2018). AMF: The future prospect for sustainable agriculture. Physiol. Mol. Plant Pathol. 102, 36–45. doi: 10.1016/j.pmpp.2017.11.007

[B13] Bauer A. A. Clayton M. K. Brunet J. (2017). Floral traits influencing plant attractiveness to three bee species: Consequences for plant reproductive success. Am. J. Bot. 104, 772–781. doi: 10.3732/ajb.1600405 28533203

[B14] Becklin K. M. Gamez G. Uelk B. Raguso R. A. Galen C. (2011). Soil fungal effects on floral signals, rewards, and aboveground interactions in an alpine pollination web. Am. J. Bot. 98, 1299–1308. doi: 10.3732/ajb.1000450 21795735

[B15] Bennett A. E. Alers-Garcia J. Bever J. D. (2006). Three-way interactions among mutualistic mycorrhizal fungi, plants, and plant enemies: Hypotheses and synthesis. Am. Nat. 167, 141–152. doi: 10.1086/499379 16670976

[B16] Bennett A. E. Meek H. C. (2020). The influence of arbuscular mycorrhizal fungi on plant reproduction. J. Chem. Ecol. 46, 707–721. doi: 10.1007/s10886-020-01192-4 32583094

[B17] Berg M. P. Ellers J. (2010). Trait plasticity in species interactions: A driving force of community dynamics. Evolutionary Ecol. 24, 617–629. doi: 10.1007/s10682-009-9347-8

[B18] Berger F. Gutjahr C. (2021). Factors affecting plant responsiveness to arbuscular mycorrhiza. Curr. Opin. Plant Biol. 59, 101994. doi: 10.1016/j.pbi.2020.101994 33450718

[B19] Berruti A. Lumini E. Balestrini R. Bianciotto V. (2016). Arbuscular mycorrhizal fungi as natural biofertilizers: Let’s benefit from past successes. Front. Microbiol. 6, 1–14. doi: 10.3389/fmicb.2015.01559 PMC4717633 26834714

[B20] Bonfante P. Anca I. A. (2009). Plants, mycorrhizal fungi, and bacteria: A network of interactions. Annu. Rev. Microbiol. 63, 363–383. doi: 10.1146/annurev.micro.091208.073504 19514845

[B21] Borowicz V. A. (2010). The impact of arbuscular mycorrhizal fungi on strawberry tolerance to root damage and drought stress. Pedobiologia 53, 265–270. doi: 10.1016/j.pedobi.2010.01.001

[B22] Bryla D. R. Koide R. T. (1998). Mycorrhizal response of two tomato genotypes relates to their ability to acquire and utilize phosphorus. Ann. Bot. 82, 849–857. doi: 10.1006/anbo.1998.0768

[B23] Burkle L. A. Runyon J. B. (2016). Drought and leaf herbivory influence floral volatiles and pollinator attraction. Global Change Biol. 22, 1644–1654. doi: 10.1111/gcb.13149 26546275

[B24] Burkle L. A. Runyon J. B. (2017). The smell of environmental change: Using floral scent to explain shifts in pollinator attraction. Appl. Plant Sci. 5, 1600123. doi: 10.3732/apps.1600123 PMC5499301 28690928

[B25] Ceuppens B. Ameye M. van Langenhove H. Roldan-Ruiz I. Smagghe G. (2015). Characterization of volatiles in strawberry varieties ‘Elsanta’ and ‘Sonata’ and their effect on bumblebee flower visiting. Arthropod-Plant Interact. 9, 281–287. doi: 10.1007/s11829-015-9375-y

[B26] Chávez M. Ferrera-Cerrato R. (1990). Effect of vesicular-arbuscular mycorrhizae on tissue culture-derived plantlets of strawberry. HortScience 25, 903–905. doi: 10.21273/HORTSCI.25.8.903

[B27] Cordeiro E. C. N. de Resende J. T. Córdova K. R. Nascimento A. Orivaldo J. S. J. Zeist A. R.. (2019). Arbuscular mycorrhizal fungi action on the quality of strawberry fruits. Horticultura Bras. 37, 437–444. doi: 10.1590/s0102-053620190412

[B28] Davison J. Moora M. Semchenko M. Adenan S. B. Ahmed T. Akhmetzhanova A. A.. (2021). Temperature and pH define the realised niche space of arbuscular mycorrhizal fungi. New Phytol. 231, 763–776. doi: 10.1111/nph.17240 33507570

[B29] De Tender C. Vandecasteele B. Verstraeten B. Ommeslag S. Kyndt T. Debode J. (2021). Biochar-enhanced resistance to Botrytis cinerea in strawberry fruits (but not leaves) is associated with changes in the rhizosphere microbiome. Front. Plant. Sci. 12, 1–14. doi: 10.3389/fpls.2021.70047910.3389/fpls.2021.700479 PMC8419269 34497619

[B30] Demirsoy H. Demirsoy L. Öztürk A. (2005). Improved model for the non-destructive estimation of strawberry leaf area. Fruits 60, 69–73. doi: 10.1051/fruits:2005014

[B31] Dumbrell A. J. Nelson M. Helgason T. Dytham C. Fitter A. H. (2010). Idiosyncrasy and overdominance in the structure of natural communities of arbuscular mycorrhizal fungi: is there a role for stochastic processes? J. Ecol. 98, 419–428. doi: 10.1111/j.1365-2745.2009.01622.x

[B32] Effmert U. Dinse C. Piechulla B. (2008). Influence of green leaf herbivory by manduca sexta on floral volatile emission by nicotiana suaveolens. Plant Physiol. 146, 1996–2007. doi: 10.1104/pp.107.112326 18281418 PMC2287366

[B33] Estaún V. Calvet C. Camprubí A. (2010). “Effect of differences among crop species and cultivars on the arbuscular mycorrhizal symbiosis,” in Arbuscular mycorrhizas: Physiology and function (279–295: Springer Netherlands).

[B34] Farré-Armengol G. Filella I. Llusià J. Peñuelas J. (2017). β-ocimene, a key floral and foliar volatile involved in multiple interactions between plants and other organisms. Molecules 22, 1148. doi: 10.3390/molecules22071148 PMC6152128 28703755

[B35] Frew A. Antunes P. M. Cameron D. D. Hartley S. E. Johnson S. N. Rillig M. C.. (2022). Plant herbivore protection by arbuscular mycorrhizas: a role for fungal diversity? New Phytol. 233, 1022–1031. doi: 10.1111/nph.17781 34618922

[B36] Gange A. C. Smith A. K. (2005). Arbuscular mycorrhizal fungi influence visitation rates of pollinating insects. Ecol. Entomology 30, 600–606. doi: 10.1111/j.0307-6946.2005.00732.x

[B37] Golubkina N. Logvinenko L. Novitsky M. Zamana S. Sokolov S. Molchanova A.. (2020). Yield, essential oil and quality performances of artemisia dracunculus, hyssopus officinalis and lavandula angustifolia as affected by arbuscular mycorrhizal fungi under organic management. Plants 9, 375. doi: 10.3390/plants9030375 PMC7154847 32197463

[B38] Grinnan R. Carter T. E. Johnson M. T. J. (2013). Effects of drought, temperature, herbivory, and genotype on plant-insect interactions in soybean (Glycine max). Arthropod-Plant Interact. 7, 201–215. doi: 10.1007/s11829-012-9234-z

[B39] Hart M. M. Antunes P. M. Chaudhary V. B. Abbott L. K. (2018). Fungal inoculants in the field: Is the reward greater than the risk? Funct. Ecol. 32, 126–135. doi: 10.1111/1365-2435.12976

[B40] Jacott C. N. Murray J. D. Ridout C. J. (2017). Trade-offs in arbuscular mycorrhizal symbiosis: Disease resistance, growth responses and perspectives for crop breeding. Agronomy 7, 75. doi: 10.3390/agronomy7040075

[B41] Janz N. (2005). The relationship between habitat selection and preference for adult and larval food resources in the polyphagous butterfly Vanessa cardui (Lepidoptera: Nymphalidae). J. Insect Behav. 18, 767–780. doi: 10.1007/s10905-005-8739-z

[B42] Kang M. S.. (1998). Using genotype-by-environment interaction for crop cultivar development. Adv. Agron. 62, 199–252. doi: 10.1016/s0065-2113(08)60569-6

[B43] Kaur S. Suseela V. (2020). Unraveling arbuscular mycorrhiza-induced changes in plant primary and secondary metabolome. Metabolites 10, 335. doi: 10.3390/metabo10080335 PMC7464697 32824704

[B44] Kelly L. Debinski D. M. (1999). Effects of larval food-limitation on Vanessa cardui Linnaeus (Lepidoptera: Nymphalidae). Am. Midland Nat. 141, 315–322. doi: 10.1674/0003-0031(1999)141[0315:EOLFLO]2.0.CO;2

[B45] Kessler A. Halitschke R. (2009). Testing the potential for conflicting selection on floral chemical traits by pollinators and herbivores: predictions and case study. Funct. Ecol. 23, 901–912. doi: 10.1111/j.1365-2435.2009.01639.x

[B46] Keymer A. Pimprikar P. Wewer V. Huber C. Brands M. Bucerius S. L.. (2017). Lipid transfer from plants to arbuscular mycorrhiza fungi. eLife 6, e29107. doi: 10.7554/eLife.29107.051 28726631 PMC5559270

[B47] Kiers T. Duhamel M. Beesetty Y. Mensah J. A. Franken O. Verbruggen E.. (2011). Reciprocal rewards stabilize cooperation I the mycorrhizal symbiosis. Science 333, 880–883. doi: 10.1126/science.1208473 21836016

[B48] Klatt B. K. Burmeister C. Westphal C. Tscharntke T. von Fragstein M. (2013). Flower volatiles, crop varieties and bee responses (N desneux, ed.). PloS One 8, e72724–e72724. doi: 10.1371/journal.pone.0072724 23977347 PMC3748066

[B49] Klatt B. K. Holzschuh A. Westphal C. Clough Y. Smit I. Pawelzik E.. (2014). Bee pollination improves crop quality, shelf life and commercial value. Proc. R. Soc. B: Biol. Sci. 281, 20132440. doi: 10.1126/science.1208473 PMC3866401 24307669

[B50] Loughrin J. H. Manukian A. Heath R. R. Turlings T. C. Tumlinson J. H. (1994). Diurnal cycle of emission of induced volatile terpenoids by herbivore-injured cotton plant. Proc. Natl. Acad. Sci. United States America 91, 11836–11840. doi: 10.1073/pnas.91.25.11836 PMC45330 11607499

[B51] Mathey M. M. Mookerjee S. Mahoney L. L. Gunduz K. Rosyara U. Hancock J. F.. (2017). Genotype by environment interactions and combining ability for strawberry families grown in diverse environments. Euphytica 213, 1-12. doi: 10.1007/s10681-017-1892-6

[B52] McCall A. C. Irwin R. E. (2006). Florivory: the intersection of pollination and herbivory. Ecol. Lett. 9, 1351–1365. doi: 10.1111/j.1461-0248.2006.00975.x 17118009

[B53] McGonigle T. P. Miller M. H. Evans D. G. Fairchild G. L. Swan J. A. (1990). A new method which gives an objective measure of colonization of roots by vesicular–arbuscular mycorrhizal fungi. New Phytol. 115, 495–501. doi: 10.1111/j.1469-8137.1990.tb00476.x 33874272

[B54] Miresmailli S. Gries R. Gries G. Zamar R. H. Isman M. B. (2010). Herbivore-induced plant volatiles allow detection of trichoplusia ni (Lepidoptera: Noctuidae) infestation on greenhouse tomato plants. Pest Manage. Sci. 66, 916–924. doi: 10.1002/ps.1967 20602512

[B55] Orians C. M. Gomez S. Korpita T. (2018). Does mycorrhizal status alter herbivore-induced changes in whole-plant resource partitioning? AoB Plants 10, 1–10. doi: 10.1093/aobpla/plx071 PMC5761529 29340134

[B56] Pagano M. C. Correa E. J. A. Duarte N. F. Yelikbayev B. O’Donovan A. Gupta V. K. (2017). Advances in eco-efficient agriculture: The plant-soil mycobiome. Agric. (Switzerland) 7, 1–12. doi: 10.3390/agriculture7020014

[B57] Pareja M. Qvarfordt E. Webster B. Mayon P. Pickett J. Birkett M.. (2012). Herbivory by a phloem-feeding insect inhibits floral volatile production. PloS One 7, e31971. doi: 10.1371/journal.pone.0031971 22384116 PMC3285634

[B58] Perner H. Schwarz D. Bruns C. Mäder P. George E. (2007). Effect of arbuscular mycorrhizal colonization and two levels of compost supply on nutrient uptake and flowering of pelargonium plants. Mycorrhiza 17, 469–474. doi: 10.1007/s00572-007-0116-7 17318595

[B59] Phillips J. Hayman D. (1970). Improved procedures for clearing roots and staining parasitic and vesicular-arbuscular mycorrhizal fungi for rapid assessment of infection. Trans. Br. Mycological Soc. 55, 158–161. doi: 10.1016/S0007-1536(70)80110-3

[B60] Raguso R. A. (2008). Wake up and smell the roses: The ecology and evolution of floral scent. Annu. Rev. Ecology Evolution Systematics 39, 549–569. doi: 10.1146/annurev.ecolsys.38.091206.095601

[B61] R Core Team (2021). R: A language and environment for statistical computing (Vienna, Austria: R foundation for Statistical Computing).

[B62] Ryan M. H. Graham J. H. (2018). Little evidence that farmers should consider abundance or diversity of arbuscular mycorrhizal fungi when managing crops. New Phytol. 220, 1092–1107. doi: 10.1111/nph.15308 29987890

[B63] Schausberger P. Peneder S. Jürschik S. Hoffmann D. (2012). Mycorrhiza changes plant volatiles to attract spider mite enemies. Funct. Ecol. 26, 441–449. doi: 10.1111/j.1365-2435.2011.01947.x

[B64] Schiestl F. P. (2010). The evolution of floral scent and insect chemical communication. Ecol. Lett. 13, 643–656. doi: 10.1111/j.1461-0248.2010.01451.x 20337694

[B65] Scott J. A. (1992). The butterflies of north America: a natural history and field guide (Stanford, California, USA: Stanford University Press).

[B66] Šimpraga M. Takabayashi J. Holopainen J. K. (2016). Language of plants: Where is the word? J. Integr. Plant Biol. 58, 343–349. doi: 10.1111/jipb.12447 26563972

[B67] Simpson D. (2018). “The economic importance of strawberry crops,” in The genomes of rosaceous berries and their wild relatives. Eds. Hytönen T. Graham J. Harrison R. (Cham: Springer International Publishing), 1–7.

[B68] Sinclair G. Charest C. Dalpé Y. Khanizadeh S. (2014). Influence of colonization by arbuscular mycorrhizal fungi on three strawberry cultivars under salty conditions. Agric. Food Sci. 23, 146–158. doi: 10.23986/afsci.9552

[B69] Smith S. E. Read D. J. (2010). Mycorrhizal symbiosis (London, UK: Elsevier Science).

[B70] Theis N. Kesler K. Adler L. S. (2009). Leaf herbivory increases floral fragrance in male but not female cucurbita pepo subsp. texana (Cucurbitaceae) flowers. Am. J. Bot. 96, 897–903. doi: 10.3732/ajb.0800300 21628242

[B71] Thirkell T. J. Charters M. D. Elliott A. J. Sait S. M. Field K. J. (2017). Are mycorrhizal fungi our sustainable saviours? considerations for achieving food security. J. Ecol. 105, 921–929. doi: 10.1111/1365-2745.12788

[B72] Todeschini V. AitLahmidi N. Mazzucco E. Marsano F. Gosetti F. Robotti E.. (2018). Impact of beneficial microorganisms on strawberry growth, fruit production, nutritional quality, and volatilome. Front. Plant Sci. 9, 1–22. doi: 10.3389/fpls.2018.01611 30505312 PMC6250784

[B73] Tooker J. F. Frank S. D. (2012). Genotypically diverse cultivar mixtures for insect pest management and increased crop yields. J. Appl. Ecol. 49, 974–985. doi: 10.1111/j.1365-2664.2012.02173.x

[B74] Torelli A. Trotta A. Acerbi L. Arcidiacono G. Berta G. Branca C. (2000). IAA and ZR content in leek (Allium porrum l.), as influenced by p nutrition and arbuscular mycorrhizae, in relation to plant development. Plant Soil 226, 29–35. doi: 10.1023/A:1026430019738

[B75] Tylianakis J. M. Didham R. K. Bascompte J. Wardle D. A. (2008). Global change and species interactions in terrestrial ecosystems. Ecol. Lett. 11, 1351–1363. doi: 10.1111/j.1461-0248.2008.01250.x 19062363

[B76] van der Putten W. H. Vet L. E. M. Harvey J. A. Wäckers F. L. (2001). Linking above- and belowground multitrophic interactions of plants, herbivores, pathogens, and their antagonists. Trends Ecol. Evol. 16, 547–554. doi: 10.1016/S0169-5347(01)02265-0

[B77] van Kleunen M. Ramponi G. Schmid B. (2004). Effects of herbivory simulated by clipping and jasmonic acid on solidago canadensis. Basic Appl. Ecol. 5, 173–181. doi: 10.1078/1439-1791-00225

[B78] Vannette R. (2020). The floral microbiome: Plant, pollinator, and microbial perspectives. Annu. Rev. Ecology Evolution Systematics 51, 363–386. doi: 10.1146/annurev-ecolsys-011720-013401

[B79] Vannette R. L. Hunter M. D. (2009). Mycorrhizal fungi as mediators of defence against insect pests in agricultural systems. Agric. For. Entomology 11, 351–358. doi: 10.1111/j.1461-9563.2009.00445.x

[B80] Varga S. Kytöviita M. M. (2010). Gender dimorphism and mycorrhizal symbiosis affect floral visitors and reproductive output in geranium sylvaticum. Funct. Ecol. 24, 750–758. doi: 10.1111/j.1365-2435.2010.01708.x

[B81] Vestberg M. (1992). Arbuscular mycorrhizal inoculation of micropropagated strawberry and field observations in Finland. Agronomie 12, 865–867. doi: 10.1051/agro:19921022

[B82] Wang M. Bezemer T. M. van der Putten W. H. Biere A. (2015). Effects of the timing of herbivory on plant defense induction and insect performance in ribwort plantain (Plantago lanceolata l.) depend on plant mycorrhizal status. J. Chem. Ecol. 41, 1006–1017. doi: 10.1007/s10886-015-0644-0 26552915 PMC4670619

[B83] Waterman J. M. Cazzonelli C. I. Hartley S. E. Johnson S. N. (2019). Simulated herbivory: The key to disentangling plant defence responses. Trends Ecol. Evol. 34, 447–458. doi: 10.1016/j.tree.2019.01.008 30824196

[B84] Wei N. Whyle R. L. Ashman T.-L. Jamieson M. A. (2022). Genotypic variation in floral volatiles influences floral microbiome more strongly than interactions with herbivores and mycorrhizae in strawberries. Horticulture Res. 9, 1-11. doi: 10.1093/hr/uhab005 PMC8795819 35141759

[B85] Wolfe B. E. Husband B. C. Klironomos J. N. (2005). Effects of a belowground mutualism on an aboveground mutualism. Ecol. Lett. 8, 218–223. doi: 10.1111/j.1461-0248.2004.00716.x

