# Supplementary material for: Genotype, mycorrhizae, and herbivory interact to shape strawberry plant functional traits
Source: Front Plant Sci. 2022 Oct 26;13:964941. doi: 10.3389/fpls.2022.964941 (PMC9644214; doi:10.3389/fpls.2022.964941)
Supplement: Supplementary file 1 [file DataSheet_1.docx]

**Supplementary Tables**

Supplementary Table 1. Summary of core strawberry floral volatile profile for each genotype (Seascape, Wasatch, Tribute). Values are means ± SD of compound emissions (ng/flower) of control plants during the ‘herbivory’ volatile sampling period during 4-hour collections.

|  | Seascape | Tribute | Wasatch |
| --- | --- | --- | --- |
| **Trans-2-hexen-1-al** | 1.94 ± 2.07 | 5.58 ± 4.27 | 30.72 ± 55.99 |
| **(E)-3-hexen-1-ol** | 22.22 ± 39.9 | 9.39 ± 7.85 | 23.71 ± 32.28 |
| **α-pinene** | 10.41 ± 19.73 | 0.74 ± 1.01 | 3.66 ± 3.5 |
| **Benzaldehyde** | 234.74 ± 102.24 | 153.02 ± 92.79 | 110.22 ± 50.36 |
| **ß-pinene** | 1.1 ± 1.99 | 0.9 ± 1.89 | 0.47 ± 0.56 |
| **6-methyl-5-hepten-2-one** | 13.54 ± 22.91 | 5.51 ± 5.83 | 9.21 ± 3.62 |
| **Cis-3-hexanyl acetate** | 148.74 ± 260.74 | 80.43 ± 44.21 | 140.46 ± 166.89 |
| **D-Limonene** | 4.29 ± 7.74 | 1.78 ± 2.59 | 3.71 ± 2.23 |
| **Benzyl alcohol** | 44.43 ± 24.06 | 17.37 ± 4.36 | 40.95 ± 5.3 |
| **Ocimene isomers** | 19.51 ± 33.19 | 11.75 ± 28.02 | 5.14 ± 3.75 |
| **Methyl salicylate** | 1.85 ± 2.05 | 4.49 ± 3.84 | 2.64 ± 2.43 |
| **Benzothiazole** | 3.49 ± 4.54 | 1.36 ± 1.21 | 1.17 ± 0.44 |
| **p-Anisaldehyde** | 117.36 ± 45.02 | 67.74 ± 36.65 | 59.69 ± 28.82 |
| **Geranyl acetate** | 1.03 ± 2.3 | 0.5 ± 0.91 | 0 |
| **Hexenyl tiglate** | 1.82 ± 4.06 | 1.15 ± 2.28 | 7.81 ± 11.72 |
| **Dihydro-ß-ionone** | 5.72 ± 7.89 | 2.79 ± 2.67 | 2.15 ± 1.82 |
| **ß-Ionone** | 5.41 ± 5.52 | 0.91 ± 1.14 | 2.8 ± 1.82 |
| **α-Muurolene** | 0.11 ± 0.25 | 1.73 ± 1.75 | 4.68 ± 7.14 |
| **α-Farnesene** | 15.93 ± 33.71 | 1.38 ± 1.37 | 4.48 ± 5.05 |
| **3-hexen-1-ol benzoate** | 1.84 ± 2.54 | 2.68 ± 5.07 | 3.02 ± 4.51 |
| **Benzyl benzoate** | 32.74 ± 16.82 | 17.88 ± 10.88 | 20.34 ± 11.93 |
| **Anisole** | 6.46 ± 6.62 | 7 ± 8.98 | 3.01 ± 1.96 |
| **Terpenes** | 77.05 ± 77.05 | 27.99 ± 27.99 | 36.3 ± 36.3 |
| **Aliphatics** | 172.9 ± 302.2 | 95.4 ± 49.06 | 194.88 ± 254.83 |
| **Benzenoids** | 439.42 ± 142.23 | 270.18 ± 134.07 | 239.89 ± 83.81 |
| **Total Emissions** | 694.68 ± 479.65 | 396.08 ± 159.43 | 480.05 ± 230.56 |

Supplementary Table 2. SIMPER (percent similarity analysis) results showing individual compound percent contributions to pairwise differences in floral volatile composition between strawberry plants receiving herbivory and control plants during the herbivory volatile sampling period. Compounds are listed in order of contribution

| **Herbivory Period** | | |
| --- | --- | --- |
| **Compound** | **Class** | **Contribution (%)** |
| ocimene isomers | Terpene | 8.95 |
| E-3-hexen-1-ol | Aliphatic | 6.88 |
| 6-methyl-5-hepten-2-one | Terpene | 6.14 |
| α-farnesene | Terpene | 5.81 |
| cis-3-hexenyl acetate | Aliphatic | 5.33 |
| trans-2-hexen-1-al | Aliphatic | 5.24 |
| α-pinene | Terpene | 5.03 |
| D-limonene | Terpene | 5.01 |
| hexenyl tiglate | C5 branched-chain | 4.49 |
| methyl salicylate | Benzenoid | 4.42 |
| benzyl benzoate | Benzenoid | 4.31 |
| 3-hexen-1-ol benzoate | Benzenoid | 4.3 |
| ß-Ionone | Terpene | 4.11 |
| dihydro-ß-ionone | Terpene | 3.92 |
| α-muurolene | Terpene | 3.91 |
| benzyl alcohol | Terpene | 3.72 |
| p-anisaldehyde | Benzenoid | 3.56 |
| anisole | Benzenoid | 3.35 |
| benzothiazole | S-containing | 3.26 |
| benzaldehyde | Benzenoid | 3.15 |
| geranyl acetate | Terpene | 2.78 |
| ß-pinene | Terpene | 2.33 |

Supplementary Table 3. SIMPER (percent similarity analysis) results showing individual compound percent contribution to pairwise differences in floral volatile composition between genotypes during the ‘recovery’ volatile sampling period. Pairwise comparisons are indicated with abbreviation, with the letter indicating strawberry genotype (S = Seascape, T = Tribute, W = Wasatch). Compounds listed in order of contribution to differences between Seascape and Tribute plants.

| **Recovery period** | | | | |
| --- | --- | --- | --- | --- |
| **Compound** | **Class** | **S-T** | **S-W** | **T-W** |
| α-pinene | terpene | 10.14 | 8.69 | 4.61 |
| ocimene isomers | terpene | 7.51 | 5.29 | 10.18 |
| α-farnesene | terpene | 5.91 | 6.28 | 3.65 |
| cis-3-hexenyl acetate | aliphatic | 5.77 | 6.49 | 6.50 |
| E-3-hexen-1-ol | aliphatic | 5.63 | 5.72 | 5.98 |
| p-anisaldehyde | benzenoid | 5.06 | 5.98 | 5.37 |
| ß-pinene | terpene | 6.08 | 4.69 | 1.73 |
| trans-2-hexen-1-al | aliphatic | 3.05 | 4.64 | 5.05 |
| hexenyl tiglate | C-5 branched-chain | 4.52 | 4.52 | 3.94 |
| D-Limonene | terpene | 4.48 | 4.96 | 5.00 |
| 6-methyl-5-hepten-2-one | terpene | 4.08 | 4.82 | 3.87 |
| α-muurolene | terpene | 4.01 | 4.07 | 3.88 |
| benzaldehyde | benzenoid | 3.85 | 3.43 | 4.26 |
| ß-ionone | terpene | 3.74 | 3.97 | 3.63 |
| benzyl benzoate | benzenoid | 3.58 | 4.74 | 5.25 |
| 3-hexen-1-ol benzoate | benzenoid | 3.57 | 2.83 | 3.68 |
| benzyl alcohol | benzenoid | 3.57 | 3.40 | 4.02 |
| dihydro-ß-ionone | terpene | 3.49 | 4.06 | 5.29 |
| methyl salicylate | benzenoid | 3.20 | 3.51 | 3.44 |
| geranyl acetate | terpene | 3.12 | 1.52 | 3.23 |
| benzothiazole | S-containing | 3.07 | 3.72 | 4.14 |
| anisole | benzenoid | 2.57 | 2.67 | 3.3 |
