# Supplementary figures and images for: Genotype, mycorrhizae, and herbivory interact to shape strawberry plant functional traits
Source: Front Plant Sci. 2022 Oct 26;13:964941. doi: 10.3389/fpls.2022.964941 (PMC9644214; doi:10.3389/fpls.2022.964941)

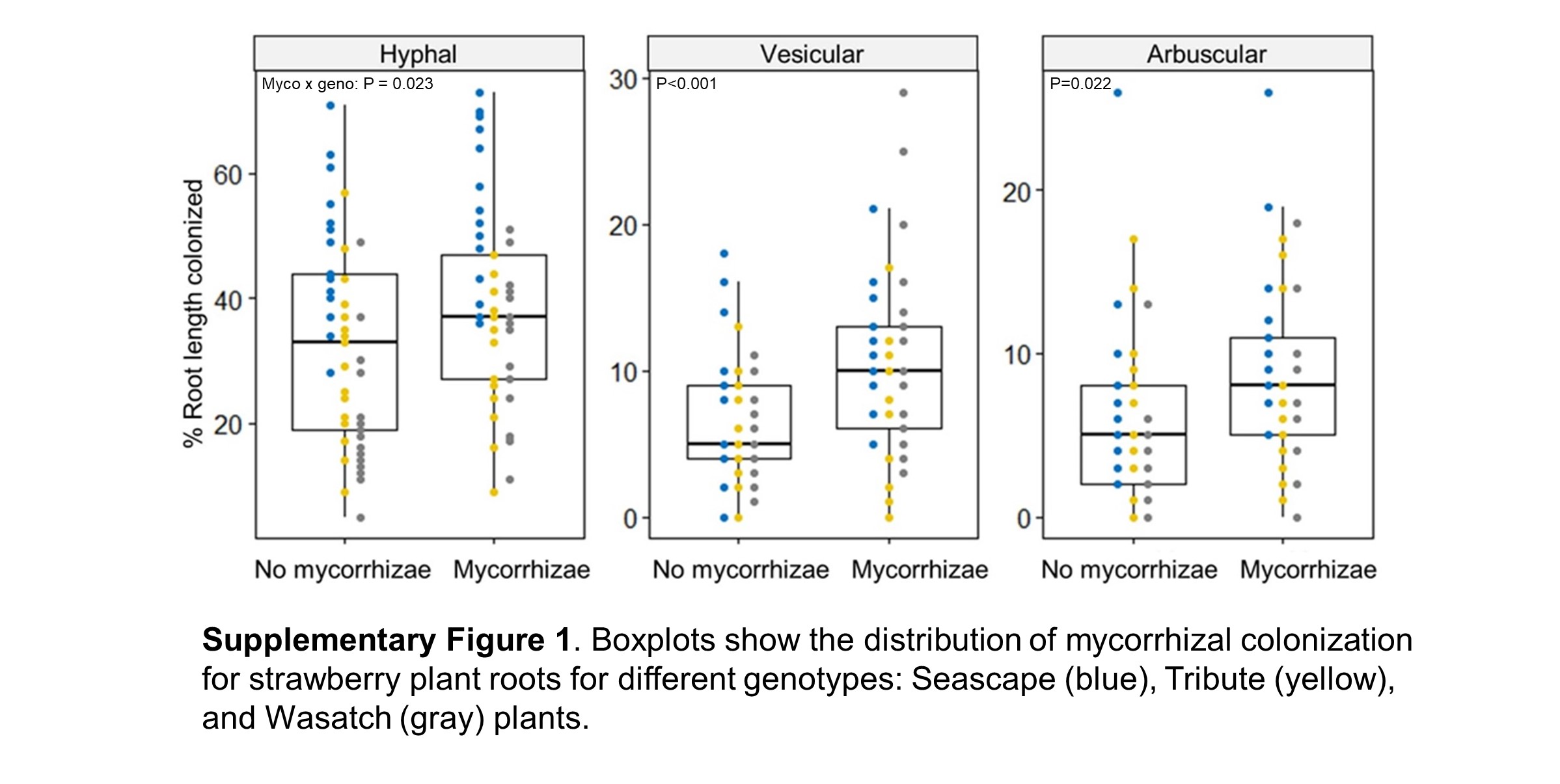

Supplement: Supplementary file 2 [file Image_1.jpeg]

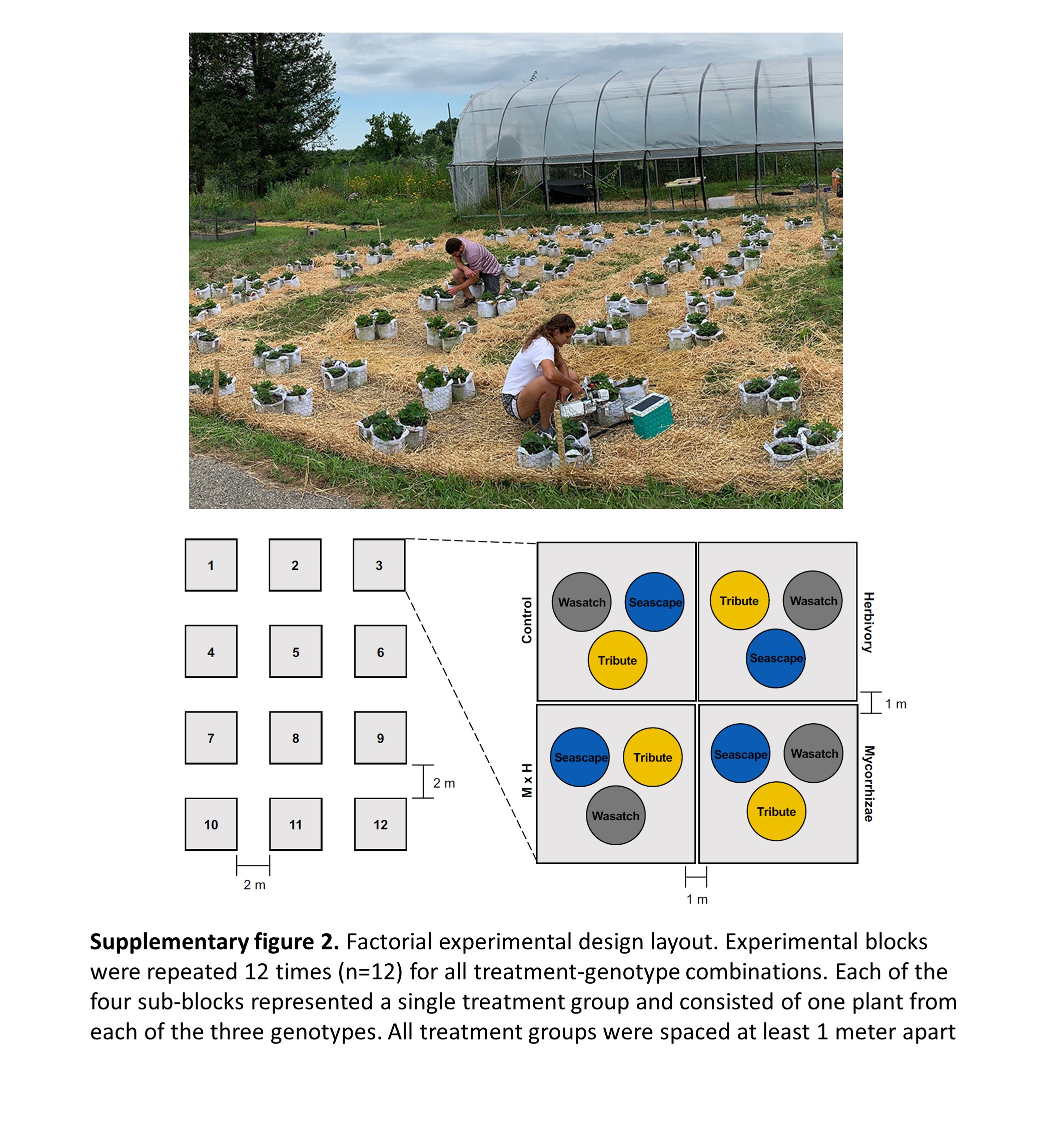

Supplement: Supplementary file 3 [file Image_2.jpeg]

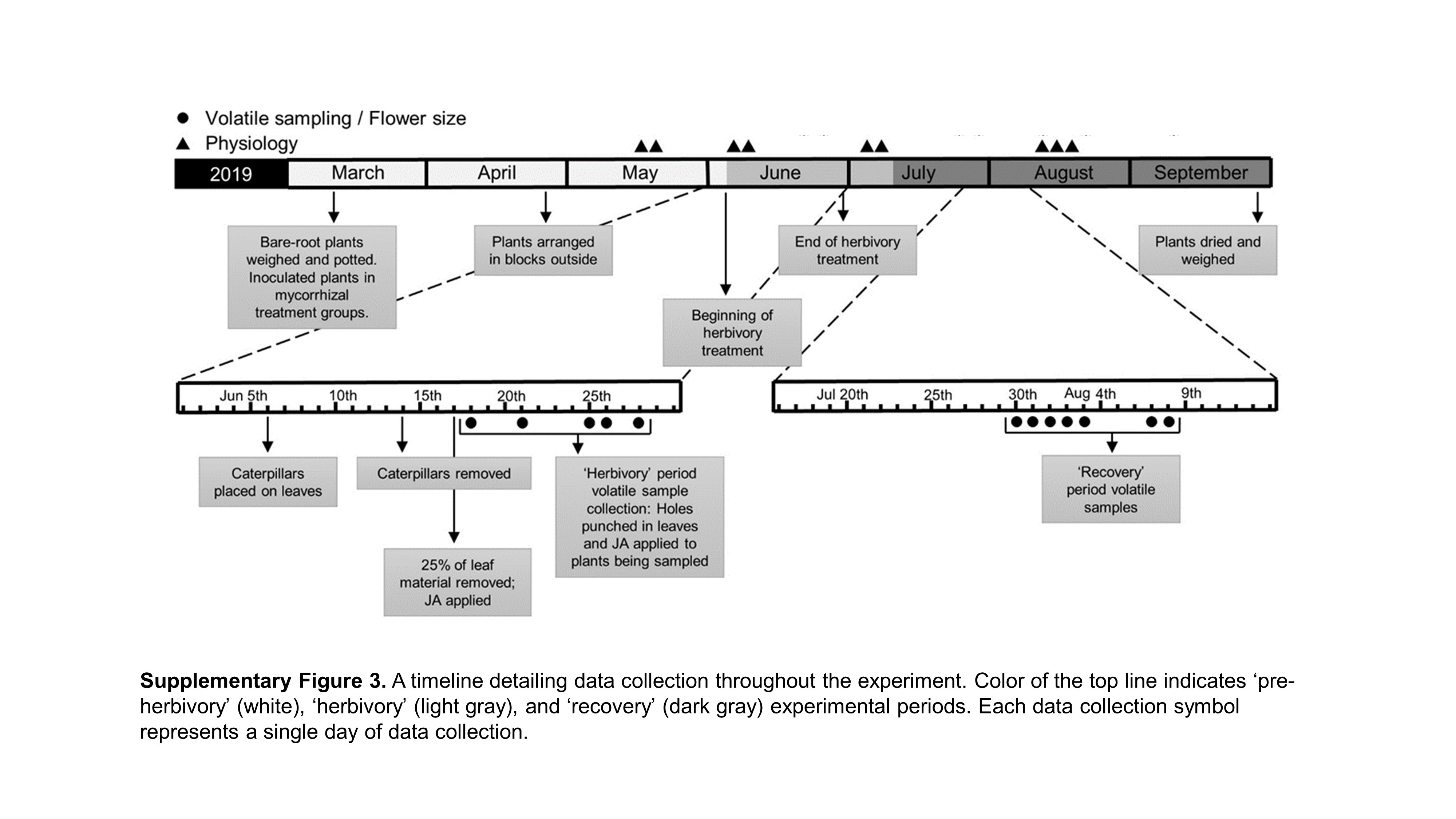

Supplement: Supplementary file 4 [file Image_3.jpeg]

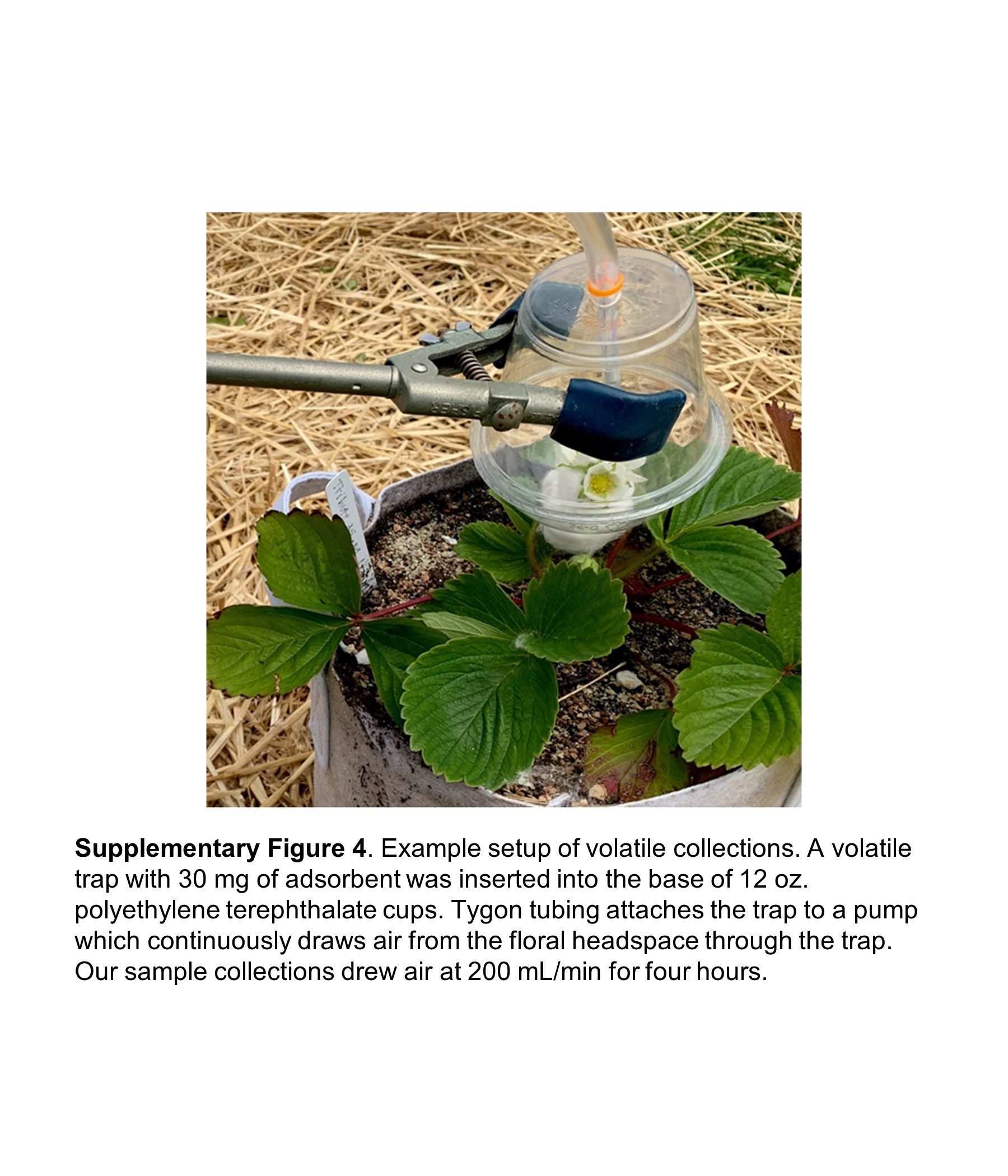

Supplement: Supplementary file 5 [file Image_4.jpeg]
